# New Polyketide Congeners with Antibacterial Activities from an Endophytic Fungus *Stemphylium globuliferum* 17035 (China General Microbiological Culture Collection Center No. 40666)

**DOI:** 10.3390/jof10110737

**Published:** 2024-10-24

**Authors:** Yingying Li, Guoliang Zhu, Jing Wang, Junjie Yu, Ke Ye, Cuiping Xing, Biao Ren, Bin Zhu, Simin Chen, Lijun Lai, Yue Li, Tom Hsiang, Lixin Zhang, Xueting Liu, Jingyu Zhang

**Affiliations:** 1 State Key Laboratory of Bioreactor Engineering, East China University of Science and Technology, Shanghai 200237, China; liyy@siyobio.com (Y.L.); zhuguoliang@ecust.edu.cn (G.Z.); wangjing_000427@163.com (J.W.); junjieyu98@outlook.com (J.Y.); y421528@163.com (K.Y.); xingcuiping123@126.com (C.X.); chensimin1205@163.com (S.C.); 18202802871@163.com (L.L.); lyhcy08042@163.com (Y.L.); lxzhang@ecust.edu.cn (L.Z.); liuxueting@ecust.edu.cn (X.L.); 2 State Key Laboratory of Oral Diseases & National Clinical Research Center for Oral Diseases, West China Hospital of Stomatology, Sichuan University, Chengdu 610041, China; renbiao@scu.edu.cn; 3 Laboratory of Pharmaceutical Crystal Engineering & Technology, Engineering Research Centre of Pharmaceutical Process Chemistry, Ministry of Education, School of Pharmacy, East China University of Science and Technology, Shanghai 200237, China; zhubin@ecust.edu.cn; 4 School of Environmental Sciences, University of Guelph, Guelph, ON N1G 2W1, Canada; thsiang@uoguelph.ca

**Keywords:** endophytic fungus, polyketides, *Stemphylium globuliferum*, antibacterial, anticancer, OSMAC strategy

## Abstract

Four new polyketides, heterocornol Y (**1**), stemphyindan (**2**), pestalospirane C (**3**), and stemphyspyrane (**4**), along with five known ones (**5**–**9**) were isolated from the endophytic fungus *Stemphylium globuliferum* 17035 (SG17035) based on the One Strain Many Compounds (OSMAC) strategy allied with an LC-MS approach. These structures were elucidated through extensive spectroscopic analyses, single-crystal X-ray diffraction, and ^13^C NMR-DP4 analysis. Pestalospirane C (**3**) and stemphyspyrane (**4**) featured unprecedented spiroketal skeletons. In addition, the putative biosynthetic logic for compounds **1**–**4** was proposed. Antibacterial and cytotoxic activities of compounds **1**–**9** were evaluated. Stemphyspyrane (**4**) displayed promising antibacterial activity against different pathogens, especially against *Staphylococcus aureus, Porphyromonas gingivalis*, and methicillin-resistant *Staphylococcus aureus* (MRSA) with MIC values of 3.125 μM, 6.25 μM, and 12.5 μM, respectively. It is promising as an antibacterial agent for further optimization.

## 1. Introduction

Fungi are one of the most important sources of bioactive natural products with enormous chemical diversity. Numerous compounds exhibit novel scaffolds with anticancer, antibacterial, antifungal, and anti-inflammatory properties and which are classified into diverse classes, such as terpenes, alkaloids, polyketones, peptides, and macrolides [1]. Moreover, many fungus-derived bioactive small molecules have been widely developed as clinical drugs and are widely used worldwide, such as penicillin, the first true antibiotic [2], lovastatin, the first lipid-lowering statin [3], and anticancer paclitaxel, which is expected to achieve mass production through plant endophytic fungi [4,5].

The term endophytes refers to a consortium of microorganisms, predominantly bacteria or fungi, that inhabit internal parts of a plant. These microorganisms reside within the host plant for the entirety or a portion of their life cycle, forming complex interactions with hosts [6,7,8]. Among various endophytes, *Stemphylium* species are well known for their production of a plethora of polyketides, including phytotoxic stemphylin, stemphyloxin I–II, and stemphyltoxin I–IV [9], cytotoxic altersolanol A–C [10,11], anti-inflammatory alterporriol Z1 and Z2 [12], antibacterial infectopyrone A, B [13], stemphyperylenol [14,15], and stemphol sulfates [16], as well as promising protein kinase inhibitors, methylalaternin, macrosporin, altersolanol A, alterporriol G, and alterporriol H [17]. In this genus, 30 species that have been identified thus far based on morphological and multi-gene phylogenic studies are also worth mentioning [18,19,20]. In addition to polyketide congeners, steroids derived from extracts of *Stemphylium* sp. MAFF 241962, an endophytic fungus from *Toona sureni*, has been found to have moderate inhibition of antimalarial activity [21]. Three novel meroterpenoids, tricycloalterfurenes E–G, were obtained from a marine-derived *Stemphylium* sp. fungus [12].

**Figure 1:**
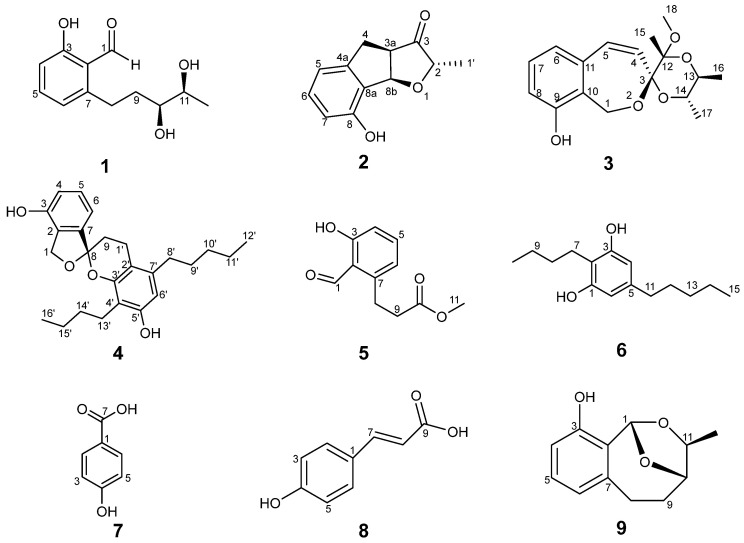
The structures of compounds **1**–**9**.

A great disparity exists when comparing the abundance of biosynthetic gene clusters (BGCs) encoding secondary metabolites and their high rediscovery rates. The discovery of natural products with architectural complexity has remained a continuous pursuit for drug development. As part of our continuous investigation into the identification of novel bioactive natural products from endophytic fungi [22,23,24], *Stemphylium globuliferum* isolated from roses has received our attention mostly because it has been reported to accumulate several polyketide monomers and dimers [17,25]. In this work, we report a series of bioactive compounds derived from *Stemphylium globuliferum* 17035 (SG17035), combining the One Strain Many Compounds (OSMAC) strategy and the LC-MS guided isolation approach. Four new polyketide derivatives named heterocornol Y (**1**), stemphyindan (**2**), pestalospirane C (**3**), and stemphyspyrane (**4**) were found together with five known compounds (Figure 1), including methyl-(2-formyl-3-hydroxyphenyl)propanoate (**5**) [26], stemphol (**6**) [16], p-hydroxybenzoic acid (**7**) [27], trans-p-hydroxy cinnamic acid (**8**) [28], and pestalotiopol C (**9**) [29]. The bioactivities of these identified compounds were evaluated against *Candida albicans*, *S*. *mutans*, *P*. *gingivalis*, *S*. *aureus*, methicillin-resistant *S*. *aureus* (MRSA), and twenty human cell lines.

## 2. Materials and Methods

### 2.1. General Experimental Procedures

Nuclear magnetic resonance (NMR) spectra (600 MHz for ^1^H and 150 MHz for ^13^C) were recorded using a Bruker Avance DRX 600 MHz spectrometer (Bruker, Billerica, MA, USA) with TCI cryoprobes. Chemical shifts were internally calibrated using the residual signals from the solvent (CDCl_3_, *δ*_H_ 7.26 and *δ*_C_ 77.0; or CD_3_OD, *δ*_H_ 2.50 and *δ*_C_ 39.5). ESIMS were conducted on an Agilent 1100 series LC/MSD (Santa Clara, CA, USA). High-resolution electrospray ionization mass spectrometry (HRESIMS) spectra were obtained on a Thermo Orbitrap Bruker Maxis II ETD QTOF mass spectrometer. Optical rotations were measured in MeOH solution on a Perkin Elmer Model 343 polarimeter (Waltham, MA, USA) with a 5 cm cell. X-ray crystallographic analysis was carried out on a Bruker APEX-III CCD diffractometer (Bruker, Billerica, MA, USA). Silica gel (100–200 mesh, 300–400 mesh, Qingdao Marine Chemical Ltd., Qingdao, China) and Sephadex LH-20 (GE Healthcare BioSciences AB, Uppsala, Sweden) were used for column chromatography (CC). Reverse-phase high-performance liquid chromatography (RP-HPLC) was carried out on an Agilent 1260 HPLC equipped with a diode array detector, and with ChemStation Rev.B.02.01 software to analyze the data. Semi-preparative RP-HPLC was conducted on a Morphling C18 column (Nanjing HeXi Biotechnology Company Limited, Nanjing, China, 10 × 250 mm, 5 μm).

### 2.2. Characterization and Identification of Fungal Strain S. globuliferum 17035 (SG17035)

The SG17035 (CGMCC: No. 40666) strain was cultured on potato dextrose agar (PDA) in an incubator (28 °C, 3 days). The ITS (internal transcribed spacer) sequence (GenBank OQ832651) and morphology were used to identify its species. The genomic DNA of SG17035 was obtained using the Rapid Fungi Genomic DNA Isolation Kit (Sangon Biotech, Shanghai, China). The primer pair ITS-F (5″-AAGGCTGGTTCCAAGACTGG-3″) and ITS-R (5″-TGGTCGTCTCTTTCGCTCCT-3″) were used to amplify the sequence from the genomic DNA by polymerase chain reaction (PCR) [23]. Afterwards, the PCR products and forward and reverse primers were sent to the Beijing Genomics Institution (BGI) for sequencing (Shanghai, China). The consensus ITS sequence was submitted to the National Center for Biotechnology Information (NCBI) for blastn analysis, and highly similar matches were obtained as well as a range of *Stemphylium* species. *Asteromyces cruciafus* was selected as the outgroup, and a phylogenetic neighbor-joining tree was built using MEGA X software with 1000 bootstrap replicates [30].

For microscopic characterization, the strain was cultured on a PDA medium plate at 28 °C for 14 days, and part of the mycelium was taken with tweezers and placed in a clean petri dish. The samples were dried using a drying oven for 24 h at 60 °C (DHP-9012, Shanghai Yiheng, Shanghai, China), coated 15 nm by a sputter coater (EM ACE200, Leica Mikrosysteme GmbH 1170, Lane Cove West, Austria), and observed under a Hitachi S-3400N scanning electron microscope (SEM, Hitachi, Tokyo, Japan).

### 2.3. Fungal Material

The culture of SG17035 was isolated from roses collected in Vancouver, Canada in 2011, and has been deposited at the China General Microbiological Culture Collection Center (accession No. 40666), Beijing, China.

### 2.4. Fermentation, Extraction, and Isolation

#### 2.4.1. Fermentation

SG17035 was cultured on plates of potato dextrose agar (PDA) [23] at 28 °C for 7 days, after which 2–3 agar blocks (0.5 × 0.5 × 0.5 cm) were placed into each of six Erlenmeyer flasks (250 mL) containing 100 mL of potato dextrose broth (PDB). After that, the Erlenmeyer flasks were incubated for 5 days at 28 °C with shaking at 200 rpm to prepare the seed culture. To find the optimal culture conditions for SG17035 based on the OSMAC strategy, 11 fungal culture media—yeast extract peptone glucose medium (GPY), T1, T7, Z4, Z5, starch, PDB, ISP2, malt extract culture medium (ME), Chashi culture medium, solid rice culture medium [23,31,32], and a blank control—were chosen for OSMAC fermentation (Table S1). After fermentation (GYP, T1, T7, Z4, Z5, starch, PDB, ISP2, ME, and Chashi for 7 days at 28 °C; solid rice culture medium for 14 days at 28 °C), an equivalent amount of ethyl acetate (EtOAc) was added and the mixture was extracted three times. Each extraction was performed with ultrasound assistance for 30 min. Following filtration, the crude fermentation extracts were obtained through vacuum distillation. Subsequently, HPLC analysis (Figure S1) of these fermentation extracts revealed that the chemical diversity on the solid rice culture medium was more abundant than on the other media. Consequently, scale-up fermentation was carried out using rice culture medium. After that, a total of 200 bags (polyethylene bag, Wuhan, China, 30 × 15 cm) of rice culture medium, each containing 80 g of Wuchang rice (Wuchang, Harbin, China) and 120 mL of deionized water, were sterilized under high pressure at 115 °C for 30 min. After cooling to room temperature, they were inoculated with 5 mL seed culture, separately, and cultured for 30 days in the dark at 28 °C to accumulate trace compounds and increase diversity of metabolites.

#### 2.4.2. Compounds Extraction and Isolation

The fermentation extracts were subjected to three successive extractions with ethyl acetate (EtOAc), and the solvent was evaporated under vacuum to obtain the crude extract (191.3 g). The crude extract was subjected to a petroleum ether (PE)/methanol (MeOH) solvent system for three successive extractions. The PE and MeOH fractions were combined separately and concentrated by rotary evaporation. The PE fraction (147.7 g) was fractionated using a silica gel column, eluting with a gradient (50:1, 20:1, 10:1, 5:1, 3:1, 1:1, PE/EtOAc) to produce twenty fractions (Frs.1–20). Sub-fraction 8 (9.6 g) was further purified through Sephadex LH-20 column chromatography (5 cm × 120 cm) eluting with MeOH-CH_2_Cl_2_ (1:1) to afford six subfractions (Frs. 8.1–8.6). Fr. 8.4 (77.8 mg) was purified using semi-preparative reverse-phase HPLC equipped with a Morphling C18 column (10 × 250 mm, 5 μm) eluting with a stepwise gradient 40–97% acetonitrile (ACN)-H_2_O (0.1% FA) for 20 min (flow rate: 3 mL/min) to obtain **5** (1.8 mg, *t*_R_ = 13.4 min) and **6** (20 mg, *t*_R_ = 18.1 min). Sub-fraction 11 (5.4 g) was subjected to reverse-phase silica gel column chromatography using a gradient MeOH–H_2_O (20:1–0:100) to yield six sub-fractions (Frs.11.1–11.6). Fr. 11.1 (45.7 mg) was further purified using semi-preparative reverse-phase HPLC (Morphling C18 column 10 × 250 mm, 5 μm, 2.5 mL/min, gradient elution with 25–35% ACN-0.1% FA/H_2_O for 25 min) to obtain **2** (1.0 mg, *t*_R_ = 13.7 min). Fr. 11.1.3 (20.0 mg) was purified using semi-preparative reverse-phase HPLC (Morphling C18 column 10 × 250 mm, 5 μm, 3 mL/min, isocratic elution with 40% ACN-0.1% FA/H_2_O for 20 min) to obtain **1** (1.01 mg, *t*_R_ = 6.2 min) and **9** (5 mg, *t*_R_ = 15.4 min). Fr. 11.3 (60.1 mg) was then fractionated using semi-preparative reverse-phase HPLC (Morphling C18 column 10 × 250 mm, 5 μm, 3.5 mL/min, gradient elution with 58–60% ACN-0.1% FA/H_2_O for 25 min) to produce **3** (1.0 mg, *t*_R_ = 12.4 min). Fr. 11.5 (1.3 g) was further purified through semi-preparative RP-HPLC (Morphling C18 column 10 × 250 mm, 5 μm, 3.5 mL/min, gradient elution with 60–85% ACN-0.1% FA/H_2_O for 20 min) to produce **4** (3.0 mg, *t*_R_ = 14.2 min).

The methanol fraction (16.6 g) was subjected to silica gel CC eluting with a gradient CH_2_Cl_2-_MeOH (100:0, 20:1, 15:1, 10:1) to produce thirteen sub-fractions (Frs.1–13). Sub-fraction 10 (310 mg) was further fractionated on Sephadex LH-20 column chromatography eluting with MeOH-CH_2_Cl_2_ (1:1) to afford eight sub-fractions (Frs. 10.1–10.8). Fr. 10.5 (22.0 mg) was further purified using semi-preparative RP-HPLC (Morphling C18 column 10 × 250 mm, 5 μm, 3.5 mL/min, gradient elution with 20–30% ACN-0.1% FA/H_2_O for 18 min) to produce **7** (2.6 mg, *t*_R_ = 9.1 min) and **8** (1.0 mg, *t*_R_ = 13.1 min).

### 2.5. Spectral Data

Heterocornol Y (**1**): white amorphous solid; ${\left[\alpha \right]}_{D}^{25}$-32.8 (c 0.5, MeOH); HRESIMS *m*/*z* 223.0968 [M-H]^−^, (calcd. for C_12_H_15_O_4_, 223.0968) (Figure S2a); UV (MeOH) λ_max_ (log ε) 220 (1.45), 265 (1.66), 340 (1.43) nm (Table 1 and Figure S2b,c).

Stemphyindan (**2**): white needle-like crystals; ${\left[\alpha \right]}_{D}^{25}$-250 (*c* 0.1, MeOH); HRESIMS *m*/*z* 205.0859 [M + H]^+^, (calcd for C_12_H_13_O_3_, 205.0862) (Figure S3a); UV (MeOH) λ_max_ (log ε) 200 (2.52), 220 (2.37) 275 (1.55) nm (Table 1 and Figure S3b,c).

Pestalospirane C (**3**): white amorphous solid; ${\left[\alpha \right]}_{D}^{25}$-48 (*c* 0.2, MeOH). HRESIMS *m*/*z* 309.1384 [M-H]^−^ (calcd. for C_17_H_21_O_5_, 305.1384) (Figure S4a); UV (MeOH) λ_max_ (log ε) 210 (2.71), 250 (2.46), 300 (1.92) nm (Table 2 and Figure S4b,c).

Stemphyspyrane (**4**): light-yellow oil; ${\left[\alpha \right]}_{D}^{25}$-58 (*c* 0.2, MeOH); HRESIMS data (*m*/*z* 395.2225 [M-H]−, calcd. for C_25_H_31_O_4_, 395.225) (Figure S5a); UV (MeOH) λ_max_ (log ε) 200 (2.22), 210 (1.69), 275 (1.49) nm (Table 1 and Figure S5b,c).

### 2.6. X-Ray Crystallographic Analysis

Compounds **1** and **2** form single crystals in methanol at 4 °C and slowly evaporate over the course of five days in an unsealed container. Single crystals of compound **3** were produced by evaporation from a methanol solution for ten days at −20 °C. A Bruker APEX-III CCD equipped with a Cu radiation source at 293 (2) K was used to gather the crystal diffraction data (Kα = 1.54178 Å). The structure was solved using the multi-scan absorption correction and OLEX2 [33] program package. The refinement was processed using the SHELXTL 5. The crystallographic data for compounds **1**–**3** has been deposited at the Cambridge Crystallographic Data Centre (CCDC) with the CCDC numbers **1**: 2377432, **2**: 2377433, and **3**: 2377434.

X-ray crystallographic data for compound **1**: C_36_H_48_O_12_ (672.74), T = 299 K, crystal system: trigonal, space group: P3_2_, *a* = 25.3482 (4) Å, *b* = 25.3482 (4) Å, *c* = 4.74190 (10) Å, *α* = 90°, *β* = 90°, *γ* = 120°, Volume 2638.62 (10) Å^3^, *Z* = 3, ρ_calc_ = 1.270 g/cm^3^, absorption coefficient: 0.786 mm^−1^, F(000) = 1080, crystal size: 0.12 × 0.09 × 0.03 mm^3^, reflections collected: 30,806, independent reflections: 6303 (*R_int_* = 0.0625), data/restraints/parameters: 6303/118/432, goodness-of-fit on F^2^: 1.065, final *R* indexes (I ≥ 2σ (I)) *R*_1_ = 0.0545, *wR*_2_ = 0.1519, *R* indices (all data): *R*_1_ = 0.0638, *wR*_2_ = 0.1621, largest diff. peak and hole: 0.369 and −0.200 e Å^−3^, Flack parameter = 0.13 (9).

X-ray crystallographic data for compound **2**: C_12_H_12_O_3_ (204.22), T = 298 K, crystal system: orthorhombic, space group: P2_1_2_1_2_1_, *a* = 4.6851 (7) Å, *b* = 9.0341 (4) Å, *c* = 24.076 (4) Å, *α* = 90°, *β* = 90°, *γ* = 90°, volume: 1019.0 (3) Å^3^, *Z* = 4, ρ_calc_ = 1.331 g/cm^3^, absorption coefficient: 0.785 mm^−1^, F(000) = 433, crystal size: 0.19 × 0.16 × 0.11 mm^3^, reflections collected: 10,974, independent reflections: 1698 (R*_int_* = 0.0746), data/restraints/parameters: 1698/0/138, goodness-of-fit on F^2^: 1.180, final *R* indexes (I ≥ 2σ (I)) *R*_1_ = 0.0487, *wR*_2_ = 0.1116, *R* indices (all data): *R*_1_ = 0.0652, *wR*_2_ = 0.1154, largest diff. peak and hole: 0.186 and −0.223 e Å^−3^, Flack parameter = 0.09(14).

X-ray crystallographic data for compound **3**: C_18_H_28_O_7_ (356.40), T = 297 K, crystal system: orthorhombic, space group: P2_1_2_1_2_1_, *a* = 7.9941 (3) Å, *b* = 10.8867 (4) Å, *c* = 21.6842 (10) Å, *α* = 90°, *β* = 90°, *γ* = 90°, volume: 1888.03 (13) Å^3^, *Z* = 4, ρ_calc_ = 1.254 g/cm^3^, absorption coefficient: 0.789 mm^−1^, F(000) = 768, crystal size: 0.16 × 0.15 × 0.11 mm^3^, reflections collected: 24,835, independent reflections: 3398 (R*_int_* = 0.0679), data/restraints/parameters: 3398/0/237, goodness-of-fit on F^2^: 1.058, final R indexes (I ≥ 2σ (I)) *R*_1_ = 0.0777, *wR*_2_ = 0.1745, *R* indices (all data): *R*_1_ = 0.0792, *wR*_2_ = 0.1769, largest diff. peak and hole: 0.480 and −0.581e Å^−3^, Flack parameter = 0.20 (7).

### 2.7. Calculation Details of ^13^C NMR and DP4 Analysis

Density functional theory (DFT), applied in Gaussian 09 [34], was used for the computations. Using the Merck Molecular Force Field (MMFF) 94 force field in Sybyl X 2.0 software with an energy cutoff value of 0.5 kcal/mol and a random search algorithm, a conformational search of all the possible isomers was conducted. Ground-state geometry was optimized at the level of B3LYP/6-31G(d). Following this, DFT geometry optimization was applied to the conformations exhibiting > 1% Boltzmann population at the B3LYP/6-311+G (d, 2p) level. With the use of both linear regression and DP4 probability, the theoretical NMR data was produced by converting the magnetic shielding values.

### 2.8. Bioactivity Tests

#### 2.8.1. General Antimicrobial Assays

Based on the antimicrobial susceptibility testing standards followed by the Clinical and Laboratory Standards Institute (CLSI) [35], the antibacterial activities of compounds **1**–**9** were assessed with the following strains of bacteria: methicillin-resistant *Staphylococcus aureus* (MRSA, No.18908, Chaoyang Hospital, Beijing, China), *Candida albicans* (SC5314), *Streptococcus mutans* (ATCC 700610), *Staphylococcus aureus* (ATCC6538), and *Porphyromonas gingivalis* (W83). Frozen glycerol stocks (−80 °C) for each organism were cultured on LB plates overnight at 37 °C. Then, for each strain, a single colony was selected and diluted to a volume of approximately 1 × 10^4^ colony-forming units (CFU)/mL in Mueller–Hinton broth (Cat. No.: CM0405B, Thermo Scientific™, Waltham, MA, USA).

Each compound was made in a two-fold dilution series in dimethyl sulfoxide (DMSO), and 2 μL was added to a 96-well flat-bottom microtiter plate which held an aliquot of the bacterial solution (78 μL) in each well. The single colony was picked up in 1 mL sterilized PBS and counted by a hemocytometer. *C*. *albicans* were 10-fold dilution in RPMI 1640 medium to approximately 1 × 10^4^ cells/mL, while other bacteria were 10-fold dilution in Mueller–Hinton broth (MHB, Cat. No.: CM0405B, Thermo Scientific™) to 1 × 10^5^ cells/mL for the further antimicrobial tests. The positive controls for the MRSA and *C*. *albicans* strains were vancomycin and amphotericin B, respectively. For the remaining strains, the antibiotic chlorhexidine was chosen as a positive control. For every strain, DMSO was chosen as a negative control. After incubation at 37 °C for 16 h, the optical density (OD) of each well at 600 nm was measured using the EnVision 2103 multi-label enzyme-linked immunosorbent assay (Perkin Elmer Life Sciences, Waltham, MA, USA). The lowest concentration of a substance that prevents a bacterium from growing visibly is known as the minimum inhibitory concentration (MIC). All the experiments were tested in triplicate.

#### 2.8.2. Cytotoxic Assays

The substances were diluted in deionized water to a concentration of 10 mM, and 20 μL were forwarded separately to respective companies that examined cytotoxic activity for preliminary screening of activity. The general experimental steps were as follows: the samples were first added to cell-grade DMSO, dissolved, and blended to a concentration of 10 μM. The concentration to be measured was ten times the concentration of the cell culture medium. Then, referring to previous articles [36,37], the CCK-8 method was used to detect the cytotoxicity of the compounds.

## 3. Results

### 3.1. Fungal Characterization and Identification

**Figure 2:**
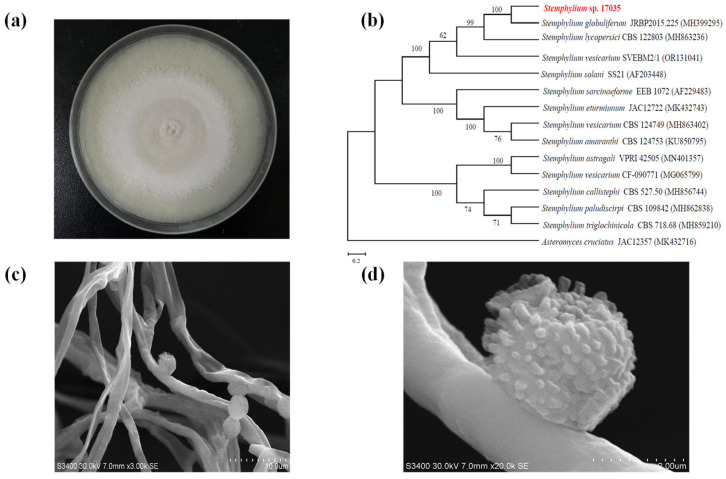
Identifying the species of strain SG17035. (**a**) SG17035’s conidiation morphology after 14 days of culture on a PDA plate at 28 °C. (**b**) The SG17035 phylogenetic tree constructed using ITS sequences. Numbers for NCBI accession are provided in parenthesis. Based on 1000 resampled datasets, numbers at nodes represent bootstrap support levels (percentages); only values > 50% are shown. The selected out-group was *Asteromyces cruciatus*. (**c**,**d**) Microscopic morphology of mycelium and conidium. Scale bars: 10 µm and 2 µm.

Morphological characteristics of strain SG17035 on a PDA plate were observed after 14 days of growth at 28 °C. The colony characteristics: cottony texture, white colonies to grayish-green with conidia maturation. Abundant mycelia growth in the central area, and a wooly halo with a white mycelial margin. Conidiophores are broadly branched (Figure 2a). A phylogenetic tree was established based on ITS sequences (Figure 2b), which showed that strain 17035 (OQ832651) and *S*. *globuliferum* JRBP 2015.255 (MH 399295) were the most similar with 100% bootstrap support. Therefore, based on morphological characteristics and phylogenetic analysis results, we identified SG17035 as *S*. *globuliferum*. The microscopic morphology of the SG17035 mycelium was examined using a scanning electron microscope (SEM). The mycelium exhibited a smooth and branched structure, with an approximate diameter of 2 µm (Figure 2c). The conidium were observed to be laterally positioned relative to the mycelium, unbranched, oval in shape, and characterized by a densely spiked surface measuring approximately 3 µm in diameter (Figure 2d), which aligned with the typical morphological features of *S*. *globuliferum* [38,39].

### 3.2. Structure Elucidation of Compounds ***1***–***9***

**Figure 3:**
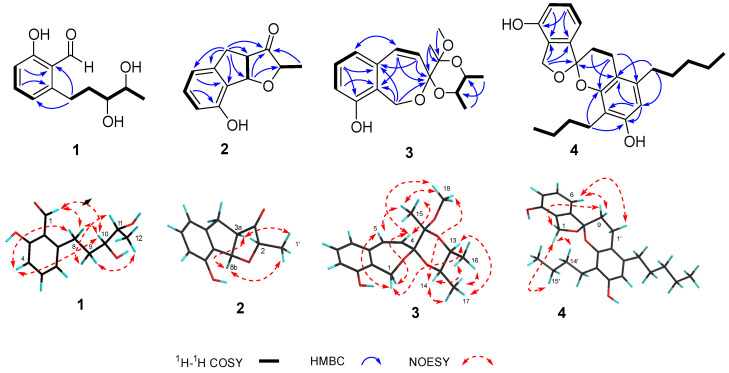
Key 2D NMR correlations of compounds **1**–**4**.

**Figure 4:**
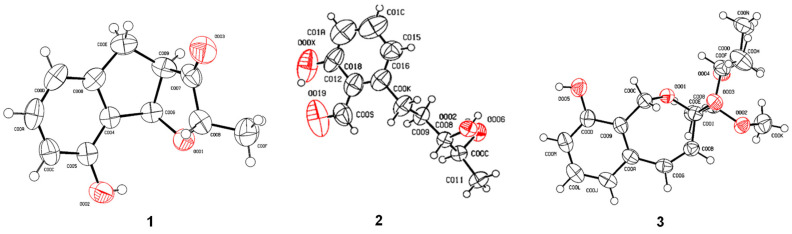
ORTEP plot (50% probability level) of single-crystal X-ray structures of **1**–**3** (red line circle: oxygen atom; black line circle: carbon atom; hollow circle: hydrogen atom).

Compound **1** was isolated as a white amorphous solid (${\left[\alpha \right]}_{D}^{25}$-32.8 (*c* 0.5, MeOH)). HRESIMS revealed a molecular ion peak of *m*/*z* 223.0968 [M-H]^−^ (calcd. for 223.0968, Figure S2a), indicating a molecular formula of C_12_H_16_O_4_. The ^1^H, ^13^C, and 2D NMR data (Table 1, Figure 3 and Figure S2b–g) of **1** showed strong agreement with those of heterocornol A [26,40], suggesting **1** as a congener. Further comparisons with the optical rotation data of the previously reported heterocornol A (${\left[\alpha \right]}_{D}^{25}$ +50.0 (*c* 0.64, MeOH)) indicated that compound **1** allowed the determination of configurations as 10*S*, 11*S*, which can be further demonstrated by single-crystal X-ray diffraction analysis as well (Figure 4). Therefore, the structure of **1** was confirmed and named as heterocornol Y (Figure 1).

Compound **2** was obtained as white needle-like crystals (${\left[\alpha \right]}_{D}^{25}$-250 (*c* 0.1, MeOH)), which has a molecular formula of C_12_H_12_O_3_ based on HRESIMS (*m*/*z* 205.0859 [M + H]^+^, calcd. for 205.0862, Figure S3a). The ^1^H and ^13^C NMR spectra of compound **2** (Table 1 and Figure S3b,c) indicated that it was a derivative of anisotindan C [41]. Comprehensive comparison of 1D and 2D NMR (Figure S3b–g) data of these two compounds revealed that one ortho-coupled aromatic group in the indane framework of anisotindan C was replaced by an aromatic hydroxyl group in compound **2**. In addition, one carbonyl carbon (*δ*_C_ 220.9) and one secondary methyl group [*δ*_C_/*δ*_H_ 16.3/1.28 (d, *J* = 6.8 Hz)] existed in the ^13^C NMR signals of compound **2**, but not in those of anisotindan C. The HMBC (Figure 3) correlation of H-6/C-8 suggested the hydroxyl group was attached to C-8. The locations of the carbonyl group at C-3 and secondary methyl group at C-2 were deduced from the HMBC correlations of H-4/C-3, H-8a/C-2, H-8b/C-3, and H_3_-1′/C-3. Moreover, this deduction was supported by X-ray (Figure 4) diffraction, and the absolute configurations of **2** were assigned as 3*R*, 11*R*, and 12*S* (Figure 1). Therefore, compound **2** was identified as an indenyl-3-keto-tetrahydrofuran derivative with a novel structure, and named stemphyindan.

Compound **3** was isolated as a white amorphous solid (${\left[\alpha \right]}_{D}^{25}$-48 (*c* 0.2, MeOH)). Its molecular formula was deduced as C_17_H_22_O_5_ on the basis of HRESIMS at *m*/*z* 305.1384 [M-H]^−^ (calcd. for 305.1384, Figure S4a). The ^1^H NMR, ^13^C NMR, ^1^H-^1^H COSY, and HSQC (Table 2, Figure S4b–e) spectra were partly consistent with those of dispiro derivatives pestalospiranes A and B [42] from C-1 to C-11, and revealed the characteristic presence of a benzo[*c*]-oxepin moiety. Observation of the two secondary methyl groups [*δ*_C_/*δ*_H_ 68.1/3.91 (m), 69.6/3.68 (m)] in the HSQC spectra (Figure S4e), in conjunction with ^1^H-^1^H COSY correlation of H-13/H-14 (Figure 3 and Figure S4d), confirmed that H-13 and H-14 were in the same spin system and verified C-13 and C-14 as attached to two oxygen atoms. Further examination on the HSQC spectra allowed the assignment of a tertiary methyl group [*δ*_C_/*δ*_H_ 17.7/1.09 (s)] and a methoxy group [*δ*_C_/*δ*_H_ 48.1/3.26 (s)]. The unprecedented 1,9,11-trioxaspiro [6.5] dodecane spiroketal skeleton was deduced from the HMBC correlations (Figure 3 and Figure S4f) of H_2_-1/C-3, H-4/C-3, H-5/C-3, and H_3_-15/C-3. The location of the methoxy group and tertiary methyl group could be determined by the HMBC correlations of H_3_-15/C-12 and H_3_-18/C-12. The location of two methyl groups at C-13 and C-14 was deduced from HMBC data as well.

The relative configurations of **3** could be further assigned by NOESY spectra (Figure 3 and Figure S4g). NOESY correlations between H-1a/H-5, H-1b/H-4, H-1b/H-13, H-4/H-13, H-4/H_3_-18, H-5/H_3_-15, and H-13/H_3_-17 revealed the relative configurations of compound **3**. Cross peak of H_2_-1a/H-13 indicated they were on the same side. In order to unambiguously assign the absolute stereochemistry of **3**, an X-ray crystal structure was obtained (Figure 4). The C-3 and C-12 atoms exhibited an *R*-configuration, and the C-13 and C-14 atoms showed an *S*-configuration. Thus, compound **3** was identified as pestalospirane C.

Compound **4** was isolated as a light-yellow oil, ${\left[\alpha \right]}_{D}^{25}$-58 (*c* 0.2, MeOH). Its molecular formula was assigned as C_25_H_32_O_4_ on the basis of HRESIMS data (*m*/*z* 395.2225 [M-H]^−^, calcd. for 395.225, Figure S5a). The ^1^H and ^13^C NMR (Figure S5b,c) for **4** revealed it is a congener of paecilospirone [43,44], that possessed an unusual architecture, spiro[chroman-2,1′(3′*H*)-isobenzofuran]. The ^1^H-^1^H COSY spectrum of **4** indicated the presence of four H-atom systems at 4-5-6, 1′-9, 8′-9′-10′-11′-12′, and 13′-14′-15′-16′ (Figure 3 and Figure S5d).

The presence of HMBC correlations (Figure 3 and Figure S5f) between H_2_-1 and H-5 with C-3 at low field confirmed the existence of a hydroxy unit at C-3. Another hydroxy group was predicted to be located at C-5′ by the HMBC correlations from H-6′ and H-13′ to C-5′. The positions of two H-atom systems 8′-9′-10′-11′-12′ and 13′-14′-15′-16′ attached to C-7′ and C-13′ were demonstrated by HMBC cross peaks from H-8′ to C-2′ and C-6′, and from H-13′ to C-3′ and C-5′, respectively. The relative configurations of **4** were assigned based on NOESY (Figure 3 and Figure S5g) correlations of H-6/H-9b, H-9a/H-1′b, H-1′b/H_2_-8′, H-6′/H_2_-8′, and H-6′/H_2_-9′. All of the signals were assigned unambiguously on the basis of 2D NMR data. In order to determine the absolute configuration at C-8, the DFT-based ^13^C NMR calculation and DP4 analysis were carried out for the 8*R* and 8*S* epimers. The results showed a higher Bayes′s theorem probability for the 8*R* configuration (100%) compared to the 8*S* configuration (0%) (Table S2). Therefore, the structure of compound **4** was confirmed as shown in Figure 1 and it was named stemphyspyrane.

**Figure 5:**
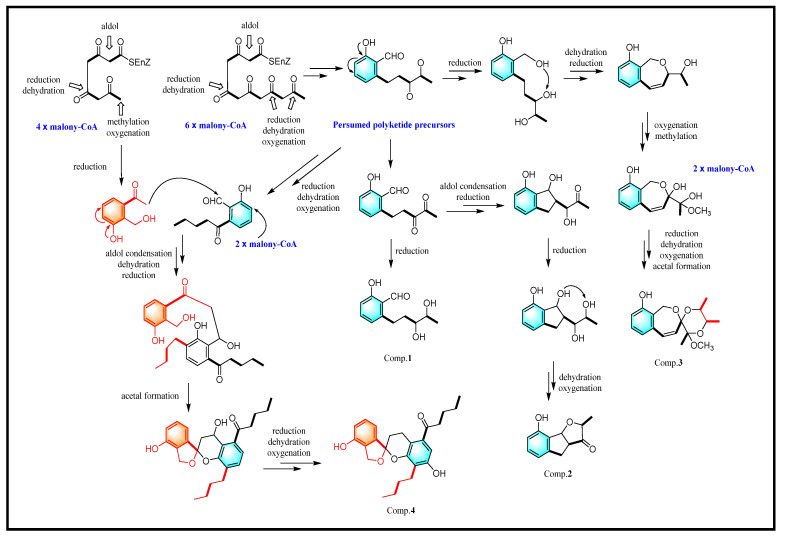
Putative biosynthetic pathway of compounds **1**–**4**.

In addition, the putative biosynthetic pathway for compounds **1**–**4** was proposed (Figure 5), and the presumed biosynthetic precursor was considered to be derived from six malonyl-CoA unites through aldol condensation, reduction, dehydration, and oxygenation [29]. Compounds **1** and **2** were biogenetically modified by successive catalyzation such as aldol condensation, reduction, oxygenation, and dehydration. Compounds **3** and **4** were finally formed by acetal formation between two presumed polyketide intermediates.

Compounds **5**–**9** were identified as methyl-(2-formyl-3-hydroxyphenyl) propanoate (**5**) [26], stemphol (**6**) [16], p-hydroxybenzoic acid (**7**) [27], trans-p-hydroxy cinnamic acid (**8**) [28], and pestalotiopol C (**9**) [29] based on their spectroscopic data and comparisons to those reported in the literature.

### 3.3. Results of Bioactivity Assay

The bioactivities of these identified compounds were evaluated against *C*. *albicans*, *S*. *mutans*, *P*. *gingivalis*, *S*. *aureus*, MRSA, and eighteen human carcinoma cell lines (Table 3 and Table S3). Compounds **2** and **3** showed weak antibacterial activity against *P*. *gingivalis* with an MIC value of 50 μM. Compound **4** exhibited activity against *S*. *aureus*, *P*. *gingivalis*, MRSA, and *S*. *mutans* with MIC values of 3.125 μM, 6.25 μM, 12.5 μM, and 50 μM. Remarkably, it did not possess obvious cytotoxicity toward normal human cell lines (Table S3). Compound **6** demonstrated activity against *P*. *gingivalis* and *S*. *aureus* with MIC values of 25 μM and 16 μM, respectively, and it exhibited mild activity against TE-1 cells with growth inhibition of 39% at 10 μM.

## 4. Discussion

Currently, the emergence of antibiotic-resistant pathogenic species represents one of the most significant challenges for drug development. Infections caused by multidrug-resistant (MDR) bacteria are increasingly prevalent, posing a critical threat to global public health. Natural products have functioned as powerful therapeutics against pathogenic bacteria since the golden age of antibiotics. The exploration of chemical entities derived from endophytic fungi is of unparalleled importance, primarily because these organisms have been recognized as a prolific source of structurally complex natural products with significant bioactive potential. Especially owing to rapid advancements in fungal genome sequencing and bioinformatics analyses of secondary metabolites, there has been a remarkable unveiling of potential for the discovery of novel natural products from endophytic fungal sources [45,46].

The OSMAC strategy has been extensively applied as an effective approach in advancing natural product discovery, facilitating the production of a diverse range of novel metabolites [47]. In an effort to enhance the chemical diversity of *S*. globuliferum 17035 (SG17035), eleven culture media were utilized to optimize its fermentation chemical profile. Our study revealed nine polyketide congeners **1**–**9** from SG17035. To date, only one report has proposed the biosynthetic pathway for compound **9** [29]. It is essential to elucidate the steps involved in the synthetic biology of these polyketides biosynthesis in further research, particularly for novel compounds, both in vitro and in vivo.

In our study, we reported antibacterial activities of compounds **1**–**9**. Notably, although compound **1** (10*S*, 11*S*) did not show relevant activity in bioactivity tests, its isomers heterocomol A (10*R*, 11*R*) and pestalotiopol A (10*R*, 11*S*) exhibited cytotoxicity against seven human cancer cell lines with IC_50_ values of 16.5–56.5 mM. Moreover, they possessed antibacterial activities against *S*. *aureus* and *Bacillus subtilis* as well, with MIC values of 25 to 100 mg/mL [26,29]. These results suggested that stereochemical configurations of hydroxyl groups at C10 and C11 may significantly influence the biological activity of the compound, particularly highlighting the importance of the *R* configuration of OH-10. Additionally, there have been no prior reports on the antibacterial activity of compounds structurally similar to **2**–**4**. Our findings represent the first evidence of their antibacterial properties, providing valuable insights into the activity diversification of polyketides. Among the known compounds **5**–**9**, only compound **6** has been isolated from *Stemphylium* sp.33231 and demonstrated significant antibacterial activity against six terrestrial pathogenic bacteria, with MIC values ranging from 0.6 to 5 µg/mL [16]. This discovery not only broadens the biological profile of these compounds but also underscores the potential for exploring other polyketide derivatives with similar frameworks in *S*. *globuliferum*.

## 5. Conclusions

In summary, utilization of the OSMAC approach efficiently extended the chemical diversity of strain SG17035 when it was cultivated on different medium, especially on solid-state fermentation with rice. This strategy allied with LC-MS guided isolation resulted in the discovery of nine polyketides (**1**–**9**), including four new compounds (**1**–**4**) and five known ones (**5**–**9**). In bioassays, compounds **2**–**4** and **6** showed antibacterial activity. Remarkably, compound **4**, which possessed a rare spiro[chroman-2,1′(3′H)-isobenzofuran] skeleton, exhibited promising anti-MASA, anti-*P*. *gingivalis*, and anti-*S*. *aureus* activities with MIC values of 12.5 µM, 6.25 μM, and 3.125 μM, respectively. Moreover, compound **6** showed weak cytotoxic activity against TE-1 cells. These data confirmed that compound **4** may be a promising lead compound serving as a foundation for the rational design and synthesis of more potent analogs.

## Figures and Tables

**Table 1:** ^l^H and^13^C NMR data of compounds **1** and **2** (*δ* in ppm, *J* in Hz).

Pos.	1		Pos.	2	
	*δ*_H_ ^a^ Mult (*J* in Hz)	*δ*_C_ ^b^, Type		*δ*_H_ ^a^ Mult (*J* in Hz)	*δ*_C_ ^b^, Type
1	10.41, s	197.3, CH	2	3.62, q (6.8)	75.6, CH
2		119.4, C	3		220.9, C
3		164.3, C	3a	3.20, m	49.3, CH
4	6.83, d (8.1)	116.6, CH	4	3.17, m 3.08, m	35.5, CH_2_
5	7.43, t (8.1)	138.3, CH	4a		145.3, C
6	6.77, d (8.1)	112.4, CH	5	6.68, d (7.8)	116.7, CH
7		148.8, C	6	7.12, t (7.8)	132.1, CH
8	3.20, ddd (13.8, 10.4, 4.9) 2.99, ddd (13.8, 10.1, 6.7)	29.0, CH_2_	7	6.65, d (7.8)	114.7, CH
9	1.80, m 1.72, dtd (14.2, 9.8, 4.8)	36.9, CH_2_	8		156.5, C
10	3.38, ddd (9.6, 4.9, 3.2)	75.6, CH	8a		127.5, C
11	3.62, qd (6.3, 4.8)	71.3, CH	8b	6.07, m	84.2, CH
12	1.13, d (6.4)	116.8, CH_3_	1′	1.28, d (6.8)	16.3, CH_3_

^a^ Recorded at 600 MHz in CDOD_3_. ^b^ Recorded at 150 MHz in CDOD_3_.

**Table 2:** ^l^H and ^13^C NMR data of compounds **3** and **4** (*δ* in ppm, *J* in Hz).

Pos.	3		Pos.	4	
	*δ*_H_ ^a^ Mult (*J* in Hz)	*δ*_C_ ^b^, Type		*δ*_H_ ^a^ Mult (*J* in Hz)	*δ*_C_ ^b^, Type
1a	4.57, d (13.7)	56.5, CH_2_	1a	5.09, d (12.9)	69.8, CH_2_
1b	5.15, d (13.7)		1b	5.22, d (12.9)	
3		100.9, C	2		126.5, C
4	6.06, d (12.7)	132.2, CH	3		149.9, C
5	6.61, d (12.7)	131.5, CH	4	6.78, d (7.8)	115.6, CH
6	6.89, d (7.7)	123.4, CH	5	7.24, t (7.8)	129.7, CH
7	7.12, t (7.7)	128.1, CH	6	6.93, d (7.8)	114.6, CH
8	6.70, d (7.7)	114.8, CH	7		143.1, C
9		152.0, C	8		108.2, C
9-OH	4.98, br		9a	2.21, ddd (13.2, 6.1, 2.3)	30.3, CH_2_
10		127.2, C	9b	2.34 td (13.2, 6.1)	
11		137.3, C	1′a	2.85 ddd (16.0, 13.0, 2.3)	18.9, CH_2_
12		99.6, C	1′b	2.99 ddd (16.0, 13.0, 6.0)	
13	3.91, m	68.1, CH	2′		112.2, C
14	3.68, m	69.6, CH	3′		151.7, C
15	1.09, s	17.7, CH_3_	4′		114.4, C
16	1.19, d (6.5)	17.2, CH_3_	5′		152.3, C
17	1.15, d (6.5)	17.1, CH_3_	6′	6.32, s	108.6, CH
18	3.26, s	48.1, CH_3_	7′		139.3, C
			8′	2.51, m	32.7, CH_2_
			9′	1.58, m	29.9, CH_2_
			10′	1.37, m	32.1, CH_2_
			11′	1.37, m	22.8, CH_2_
			12′	0.92, t (7.3)	14.2, CH_3_
			13′	2.45, t (7.4)	22.6, CH_2_
			14′	1.37, m	31.6, CH_2_
			15′	1.22, m	22.5, CH_2_
			16′	0.76, t (7.3)	14.0, CH_3_

^a^ Recorded at 600 MHz in CDCl_3_. ^b^ Recorded at 150 MHz in CDCl_3_.

**Table 3:** Antimicrobial activity of **1**–**9**.

Compound	Pathogenic Bacteria (MIC, μM)				
	*C. albicans*	*S. mutans*	*P. gingivalis*	*S. aureus*	MRSA
1	>100	>100	>100	>100	>100
2	>100	>100	50	>100	>100
3	>100	>100	50	>100	>100
4	>100	50	6.25	3.125	12.5
5	>100	>100	>100	>100	-
6	100	100	25	16	-
7	>100	>100	>100	>100	-
8	>100	>100	>100	>100	-
9	>100	>100	>100	>100	-
Amphotericin B ^a^	1	-	-	-	-
Chlorhexidine ^b^	-	2	1	1	-
Vancomycin ^c^	-	-	-	-	1

^a^ Amphotericin B was used as a positive control for *C. albicans* (SC5314); ^b^ chlorhexidine was used as a positive control for *S. mutans* (ATCC700610), *P. gingivalis* (W83), and *S. aureus* (ATCC6538); ^c^ vancomycin was used as a positive control for MRSA (No. 18908); each value was expressed as a mean ± standard deviation (*n* = 3).

## Data Availability

The original contributions presented in the study are included in the article/Supplementary Materials, further inquiries can be directed to the corresponding author.

## Supplementary Materials

The following supporting information can be downloaded at: https://www.mdpi.com/article/10.3390/jof10110737/s1, Figure S1. RP-HPLC chromatograms of (a) the SG 17035 fermented in eleven different culture media using OSMAC strategy detected at 210 nm, and (b) the SG 17035 fermented on rice solid culture media (210 nm) including the identification of compounds **1–9**; Figure S2. (a) HRESIMS spectrum of **1**; (b) ^1^H NMR spectrum (600 MHz, CD_3_OD) of **1**; (c) ^13^C NMR spectrum (150 MHz, CD_3_OD) of **1**; (d) ^1^H-^1^H COSY spectrum (600 MHz, CD_3_OD) of **1**; (e) HSQC spectrum (150 MHz/600 MHz, CD_3_OD) of **1**; (f) HMBC spectrum (150 MHz/600 MHz, CD_3_OD) of **1**; (g) NOESY spectrum (600 MHz, CD_3_OD) of **1**; Figure S3. (a) HRESIMS spectrum of **2**; (b) ^1^H NMR spectrum (600 MHz, CD_3_OD) of **2**; (c) ^13^C NMR spectrum (150 MHz, CD_3_OD) of **2**; (d) ^1^H-^1^H COSY spectrum (600 MHz, CD_3_OD) of **2**; (e) HSQC spectrum (150 MHz/600 MHz, CD_3_OD) of **2**; (f) HMBC spectrum (150 MHz/600 MHz, CD_3_OD) of **2**; (g) NOESY spectrum (600 MHz, CD_3_OD) of **2**; Figure S4. (a) HRESIMS spectrum of **3**; (b) ^1^H NMR spectrum (600 MHz, CDCl_3_) of **3**; (c) ^13^C NMR spectrum (150 MHz, CDCl_3_) of **3**; (d) ^1^H-^1^H COSY spectrum (600 MHz, CDCl_3_) of **3**; (e) HSQC spectrum (150 MHz/600 MHz, CDCl_3_) of **3**; (f) HMBC spectrum (150 MHz/600 MHz, CDCl_3_) of **3**; (g) NOESY spectrum (600 MHz, CDCl_3_) of **3**; Figure S5. (a) HRESIMS spectrum of **4**; (b) ^1^H NMR spectrum (600 MHz, CDCl_3_) of **4**; (c) ^13^C NMR spectrum (150 MHz, CDCl_3_) of **4**; (d) ^1^H-^1^H COSY spectrum (600 MHz, CDCl_3_) of **4**; (e) HSQC spectrum (150 MHz/600 MHz, CDCl_3_) of **4**; (f) HMBC spectrum (150 MHz/600 MHz, CDCl_3_) of **4**; (g) NOESY spectrum (600 MHz, CDCl_3_) of **4**; Figure S6. (a) HRESIMS spectrum of **5**; (b) ^1^H NMR spectrum (600 MHz, CDCl_3_) of **5**; (c) ^13^C NMR spectrum (150 MHz, CDCl_3_) of **5**; Figure S7. (a) HRESIMS spectrum of **6**; (b) ^1^H NMR spectrum (600 MHz, CDCl_3_) of **6**; (c) ^13^C NMR spectrum (150 MHz, CDCl_3_) of **6**; Figure S8. (a) HRESIMS spectrum of **7**; (b) ^1^H NMR spectrum (600 MHz, CD_3_OD) of **7**; Figure S9. (a) HRESIMS spectrum of **8**; (b) ^1^H NMR spectrum (600 MHz, CD_3_OD) of **8**; (c) ^13^C NMR spectrum (150 MHz, CD_3_OD) of **8**; Figure S10. (a) HRESIMS spectrum of **9**; (b) ^1^H NMR spectrum (600 MHz, CD_3_OD) of **9**; (c) ^13^C NMR spectrum (150 MHz, CD_3_OD) of **9**; Table S1: Composition of the culture media; Table S2: DP4 probability of C NMR chemical shifts of 4a (**8***R*) and 4b (**8***S*); Table S3: Cytotoxic activity of **1**–**9**.
