# Supplementary material for: New Polyketide Congeners with Antibacterial Activities from an Endophytic Fungus Stemphylium globuliferum 17035 (China General Microbiological Culture Collection Center No. 40666)
Source: J Fungi (Basel). 2024 Oct 24;10(11):737. doi: 10.3390/jof10110737 (PMC11595617; doi:10.3390/jof10110737)
Supplement: Supplementary file 1 [file jof-10-00737-s001.zip › jof-3226252-supplementary.pdf]

Supporting Information

New Polyketide Congeners with Antibacterial Activities from an Endophytic Fungus *Stemphylium globuliferum* 17035 (China General Microbiological Culture Collection Center No. 40666)

Yingying Li [1], Guoliang Zhu [1], Jing Wang [1], Junjie Yu [1], Ke Ye [1], Cuiping Xing [1], Biao Ren [2], Bin Zhu [3], Simin Chen [1], Lijun Lai [1], Yue Li [1], Tom Hsiang [4], Lixin Zhang [1], Xueting Liu [1] and Jingyu Zhang [1,*]

[1] State Key Laboratory of Bioreactor Engineering, East China University of Science and Technology, Shanghai 200237, China; liyy@siyobio.com (Y.L.); zhuguoliang@ecust.edu.cn (G.Z.); wangjing_000427@163.com (J.W.); junjieyu98@outlook.com (J.Y.); y421528@163.com (K.Y.); xingcuiping123@126.com (C.X.); chensimin1205@163.com (S.C.); 18202802871@163.com (L.L.); lyhcy08042@163.com (Y.L.); lxzhang@ecust.edu.cn (L.Z.); liuxueting@ecust.edu.cn (X.L.)
[2] State Key Laboratory of Oral Diseases & National Clinical Research Center for Oral Diseases, West China Hospital of Stomatology, Sichuan University, Chengdu 610041, China; renbiao@scu.edu.cn
[3] Laboratory of Pharmaceutical Crystal Engineering & Technology, Engineering Research Centre of Pharmaceutical Process Chemistry, Ministry of Education, School of Pharmacy, East China University of Science and Technology, Shanghai 200237, China; zhubin@ecust.edu.cn
[4] School of Environmental Sciences, University of Guelph, Guelph, ON N1G 2W1, Canada; thsiang@uoguelph.ca

* Correspondence: zhangjingyu@ecust.edu.cn; Tel.: +86-21-64253020

Table of contents

Supplementary Figures 5

Figure S1. RP-HPLC chromatograms of (**a**) the SG 17035 fermented in eleven...... 5 different culture media using OSMAC strategy detected at 210 nm, and (**b**) the SG 17035 fermented on rice solid culture medium (210 nm) including the identification of compounds **1-9**... 5

Figure S2a HRESIMS spectrum of **1**... 6

Figure S2b ^{1}H NMR spectrum (600 MHz, CD_3OD) of **1**................................ 6

Figure S2c ^{13}C NMR spectrum (150 MHz, CD_3OD) of **1**. 7

Figure S2d ^{1}H-^{1}H COSY spectrum (600 MHz, CD_3OD) of **1**. 7

Figure S2e HSQC spectrum (150 MHz/600 MHz, CD_3OD) of **1**........................ 8

Figure S2f HMBC spectrum (150 MHz/600 MHz, CD_3OD) of **1**. 8

Figure S2g NOESY spectrum (600 MHz, CD_3OD) of **1**.................................. 9

Figure S3a HRESIMS spectrum of **2**... 9

Figure S3b ^{1}H NMR spectrum (600 MHz, CD_3OD) of **2**.............................. 10

Figure S3c ^{13}C NMR spectrum (150 MHz, CD_3OD) of **2**. 10

Figure S3d ^{1}H-^{1}H COSY spectrum (600 MHz, CD_3OD) of **2**. 11

Figure S3e HSQC spectrum (150 MHz/600 MHz, CD_3OD) of **2**...................... 11

Figure S3f HMBC spectrum (150 MHz/600 MHz, CD_3OD) of **2**. 12

Figure S3g NOESY spectrum (600 MHz, CD_3OD) of **2**................................ 12

Figure S4a HRESIMS spectrum of **3**... 13

Figure S4b ^{1}H NMR spectrum (600 MHz, $CDCl_3$) of **3**. 13

Figure S4c ^{13}C NMR spectrum (150 MHz, $CDCl_3$) of **3**............................... 14

Figure S4d ^{1}H-^{1}H COSY spectrum (600 MHz, $CDCl_3$) of **3**. 14

Figure S4e HSQC spectrum (150 MHz/600 MHz, $CDCl_3$) of **3**. 15

Figure S4f HMBC spectrum (150 MHz/600 MHz, $CDCl_3$) of **3**. 15

Figure S4g NOESY spectrum (600 MHz, $CDCl_3$) of **3**. 16

Figure S5a HRESIMS spectrum of **4**. 16

Figure S5b ^{1}H NMR spectrum (600 MHz, $CDCl_3$) of **4**. 17

Figure S5c ^{13}C NMR spectrum (150 MHz, $CDCl_3$) of **4**. 17

Figure S5d ^{1}H-^{1}H COSY spectrum (600 MHz, $CDCl_3$) of **4**. 18

Figure S5e HSQC spectrum (150 MHz/600 MHz, $CDCl_3$) of **4**. 18

Figure S5f HMBC spectrum (150 MHz/600 MHz, $CDCl_3$) of **4**. 19

Figure S5g NOESY spectrum (600 MHz, $CDCl_3$) of **4**. 19

Figure S6a HRESIMS spectrum of **5**. 20

Figure S6b ^{1}H NMR spectrum (600 MHz, $CDCl_3$) of **5**. 20

Figure S6c ^{13}C NMR spectrum (150 MHz, $CDCl_3$) of **5**. 21

Figure S7a HRESIMS spectrum of **6**. 21

Figure S7b ^{1}H NMR spectrum (600 MHz, $CDCl_3$) of **6**. 22

Figure S7c ^{13}C NMR spectrum (150 MHz, $CDCl_3$) of **6**. 22

Figure S8a HRESIMS spectrum of **7**. 23

Figure S8b ^{1}H NMR spectrum (600 MHz, CD_3OD) of **7**. 23

Figure S9a HRESIMS spectrum of **8**. 24

Figure S9b ^{1}H NMR spectrum (600 MHz, CD_3OD) of **8**. 24

Figure S9c ^{13}C NMR spectrum (150 MHz, CD_3OD) of **8**. 25

Figure S10a HRESIMS spectrum of **9**. 25

Figure S10b ^{1}H NMR spectrum (600 MHz, CD_3OD) of **9**. 26

Figure S10c ^{13}C NMR spectrum (150 MHz, CD_3OD) of **9**............................. 26

Supplementary Tables 27

Table S1. Composition of the culture media.. 27

Table S2. DP4 probability of C NMR chemical shifts of **4a** (8*R*) and **4b** (8*S*)....... 28

Table S3. Cytotoxic activity of **1−9** 29

Supplementary Figures

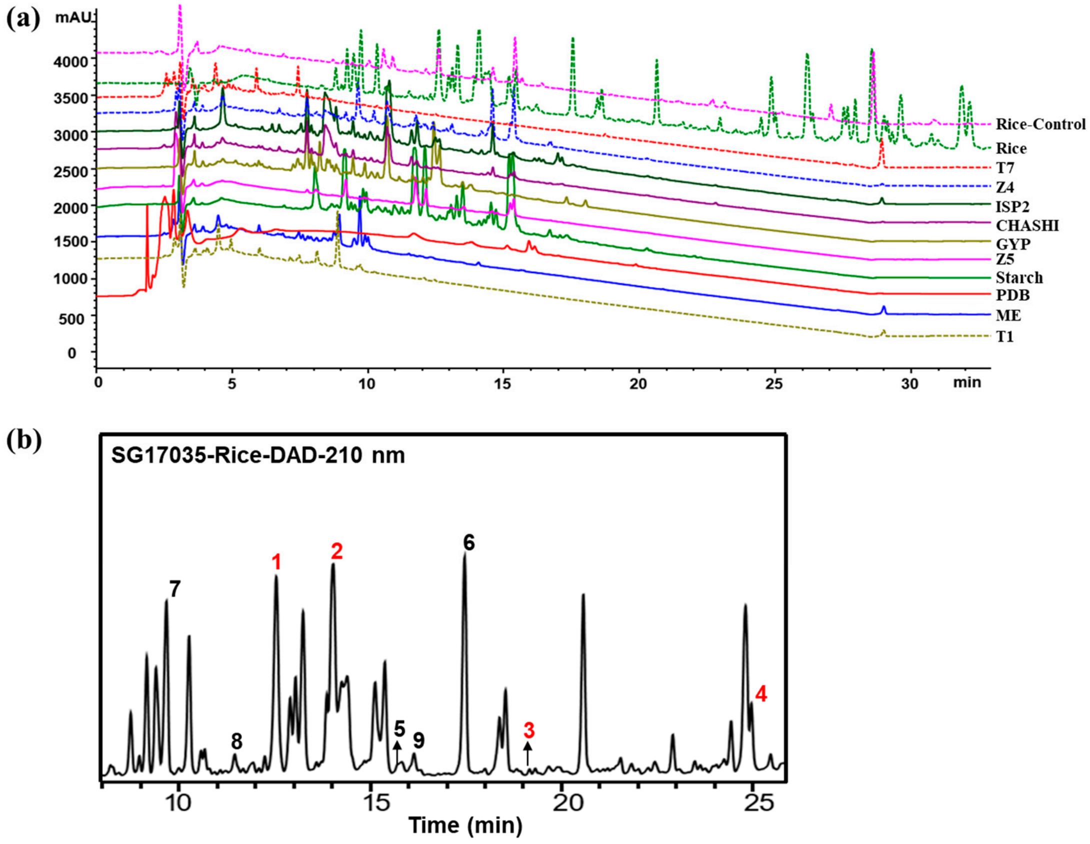

Figure S1. RP-HPLC chromatograms of (**a**) the SG 17035 fermented in eleven different culture media using OSMAC strategy detected at 210 nm, and (**b**) the SG 17035 fermented on rice solid culture medium (210 nm) including the identification of compounds **1-9**.

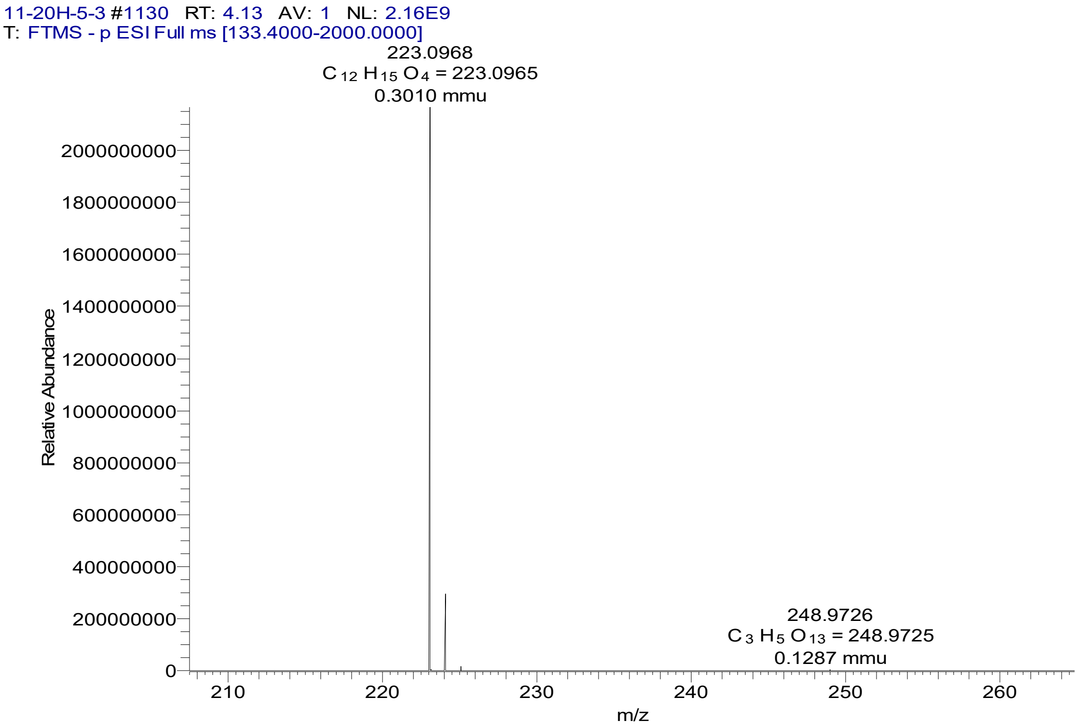

Figure S2a HRESIMS spectrum of **1**.

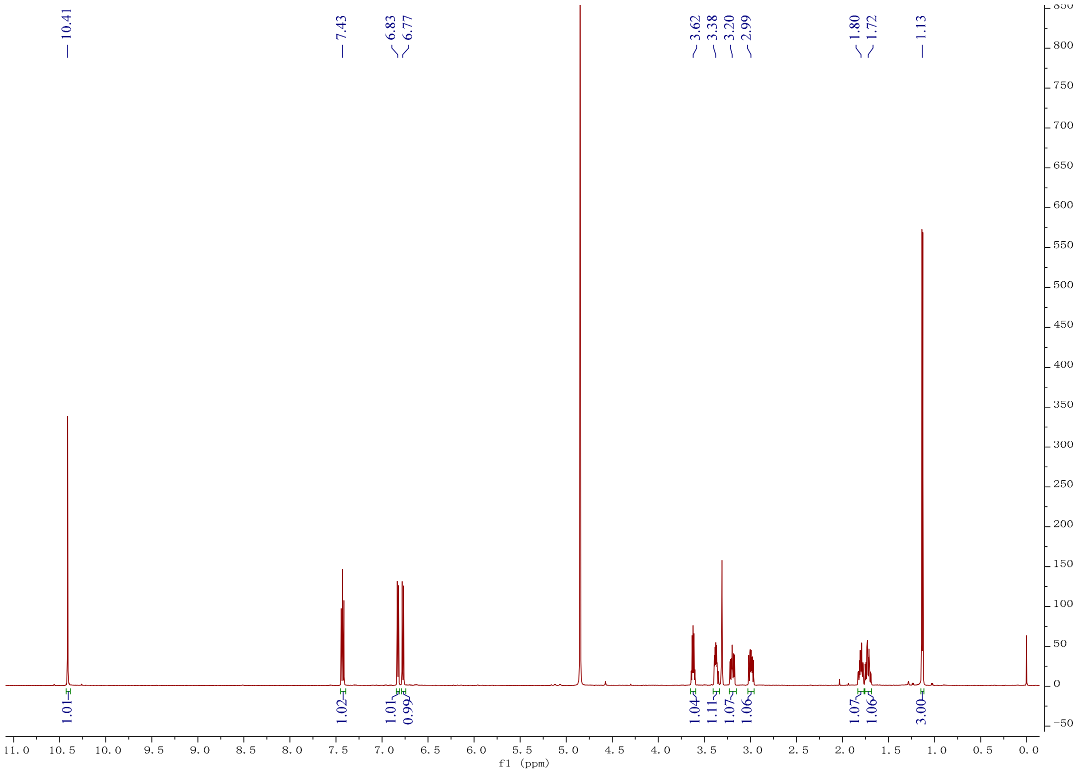

Figure S2b ^{1}H NMR spectrum (600 MHz, CD_3OD) of **1**.

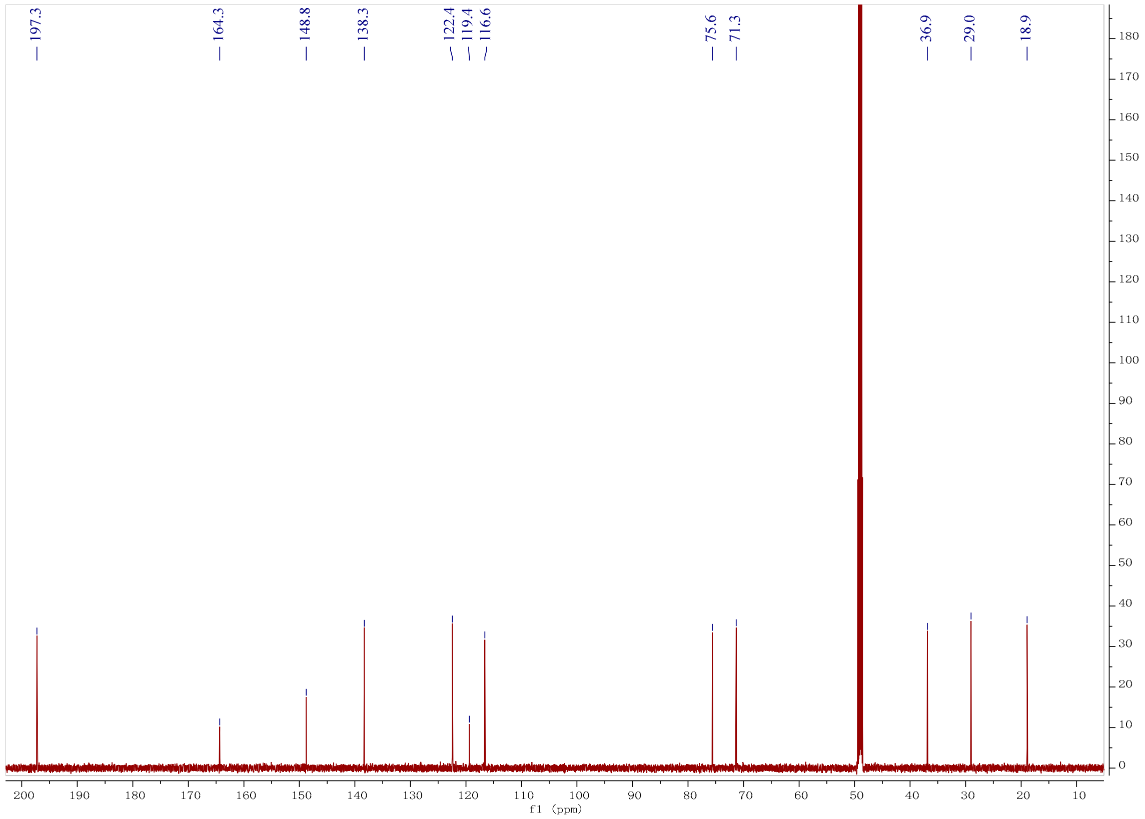

Figure S2c ^{13}C NMR spectrum (150 MHz, CD_3OD) of **1**.

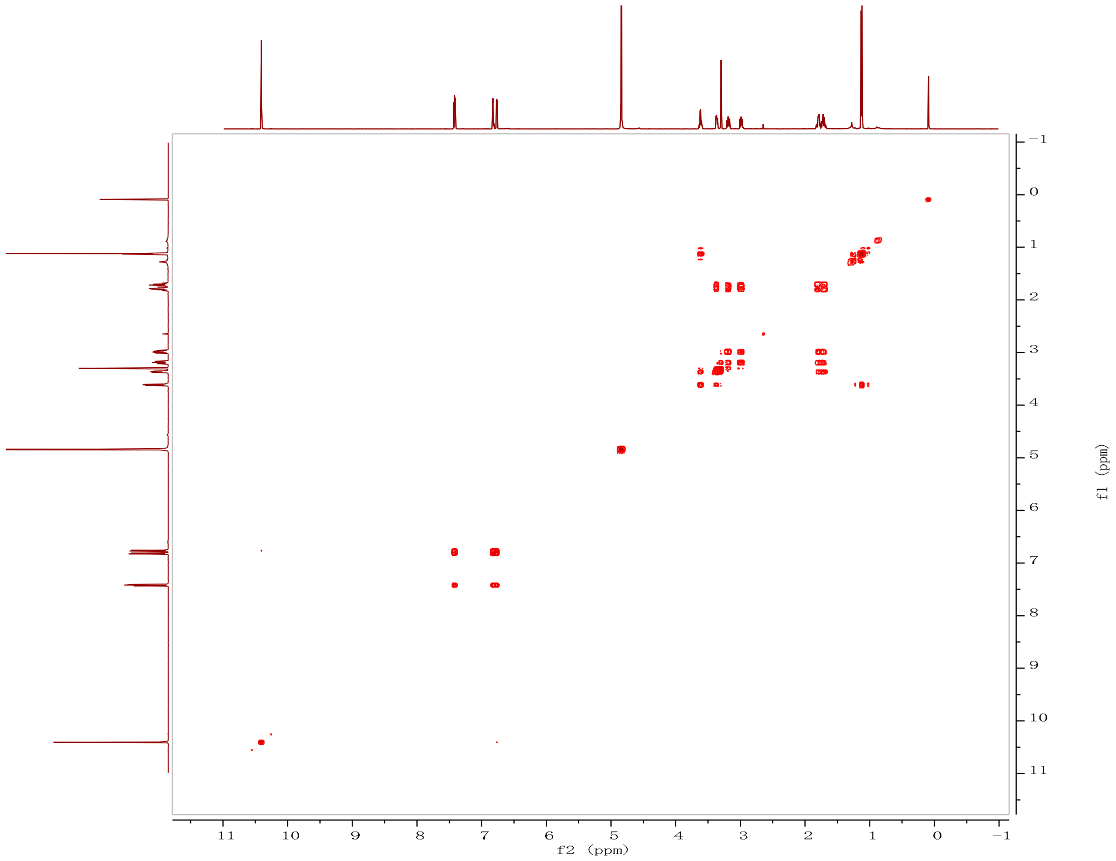

Figure S2d ^{1}H-^{1}H COSY spectrum (600 MHz, CD_3OD) of **1**.

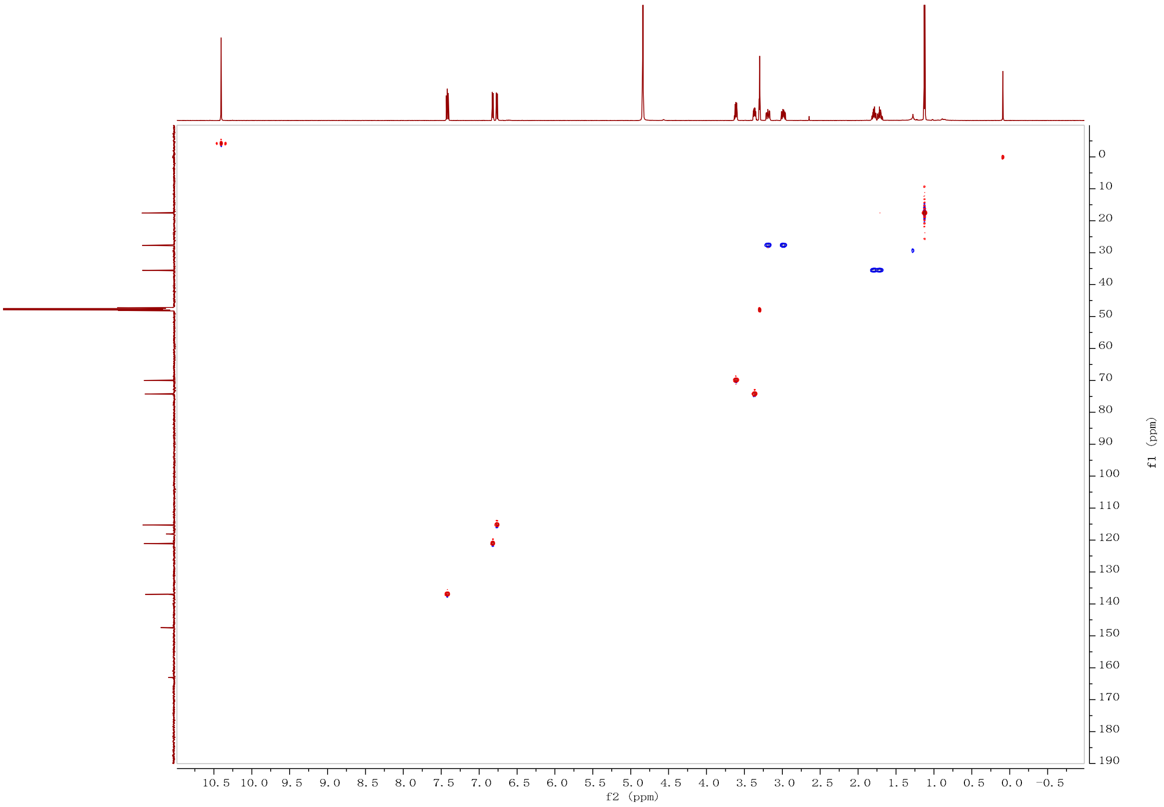

Figure S2e HSQC spectrum (150 MHz/600 MHz, CD3OD) of **1**.

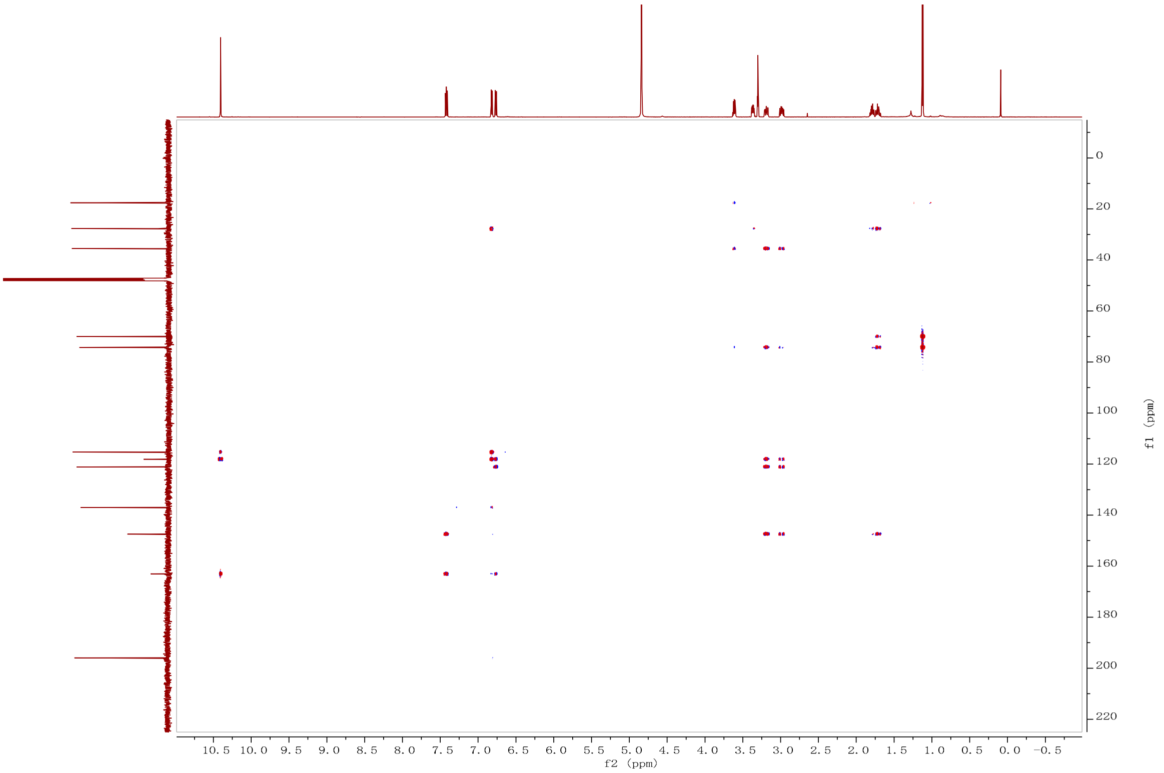

Figure S2f HMBC spectrum (150 MHz/600 MHz, CD3OD) of **1**.

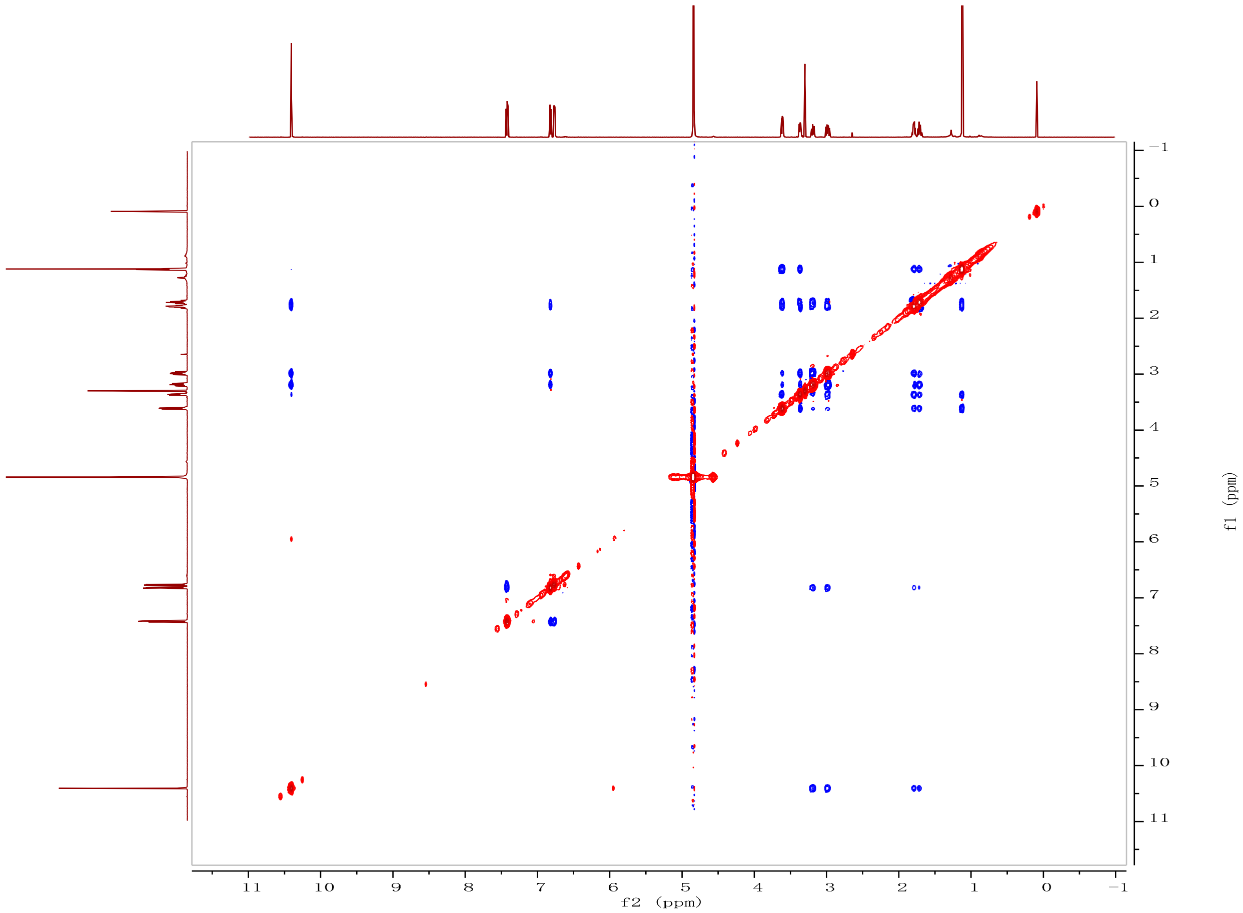

Figure S2g NOESY spectrum (600 MHz, CD_3OD) of **1**.

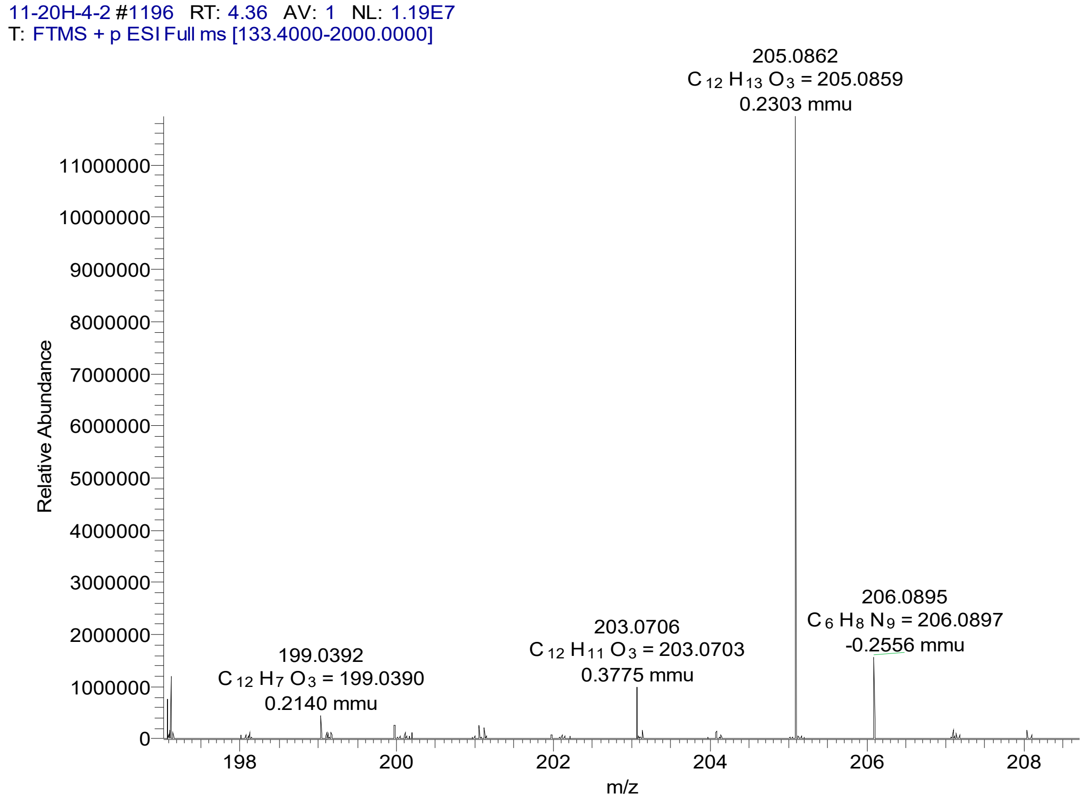

Figure S3a HRESIMS spectrum of **2**.

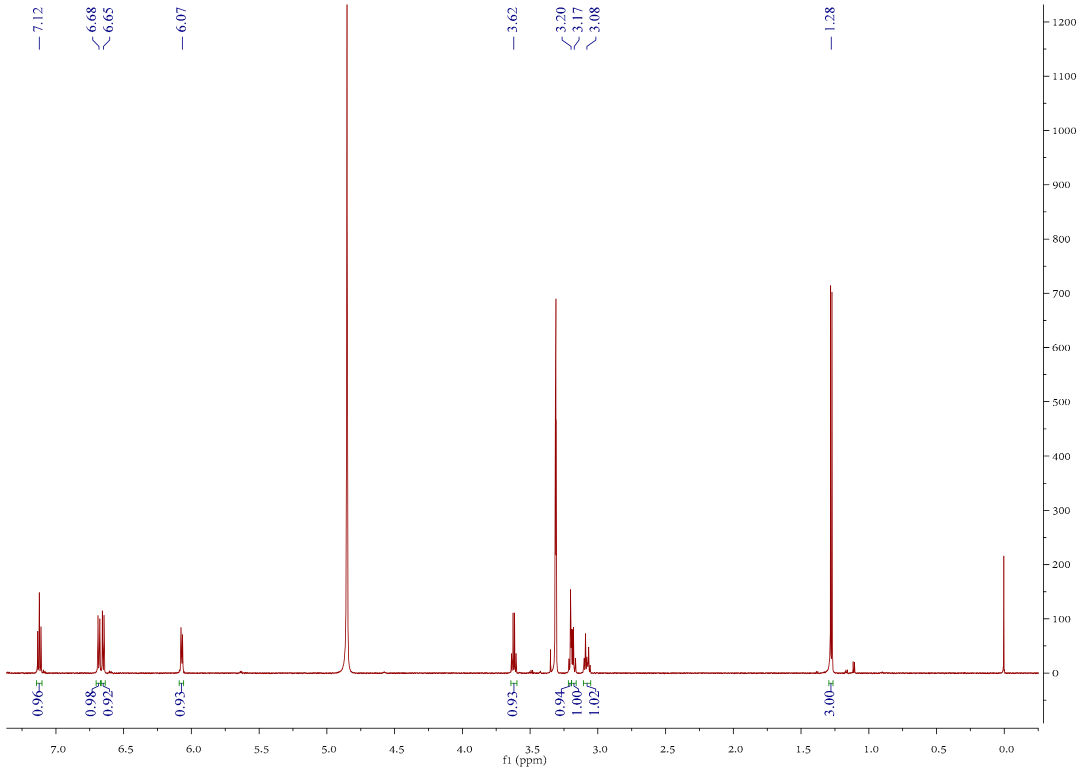

Figure S3b ^{1}H NMR spectrum (600 MHz, CD_3OD) of **2**.

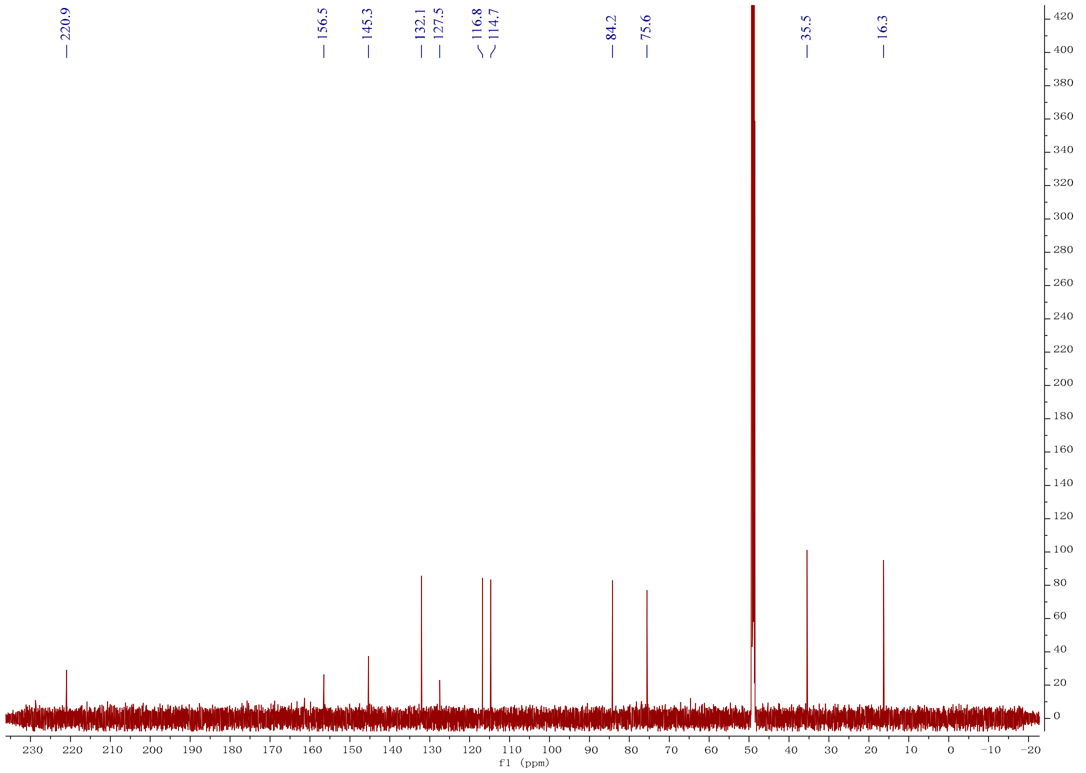

Figure S3c ^{13}C NMR spectrum (150 MHz, CD_3OD) of **2**.

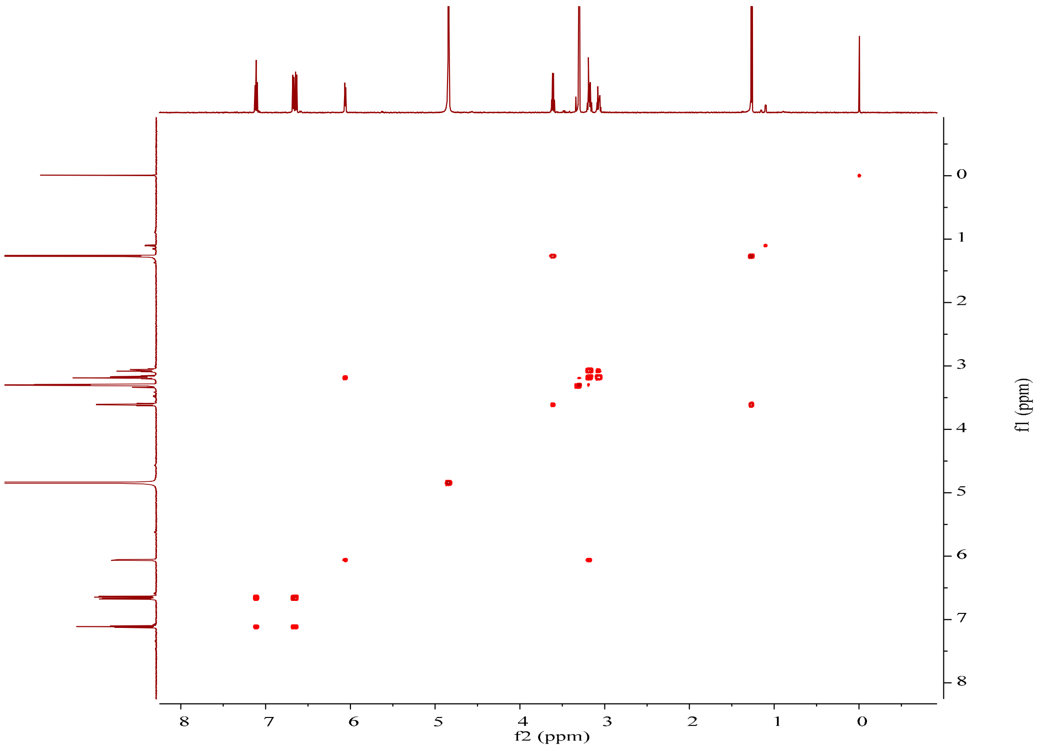

Figure S3d ^{1}H-^{1}H COSY spectrum (600 MHz, CD_3OD) of **2**.

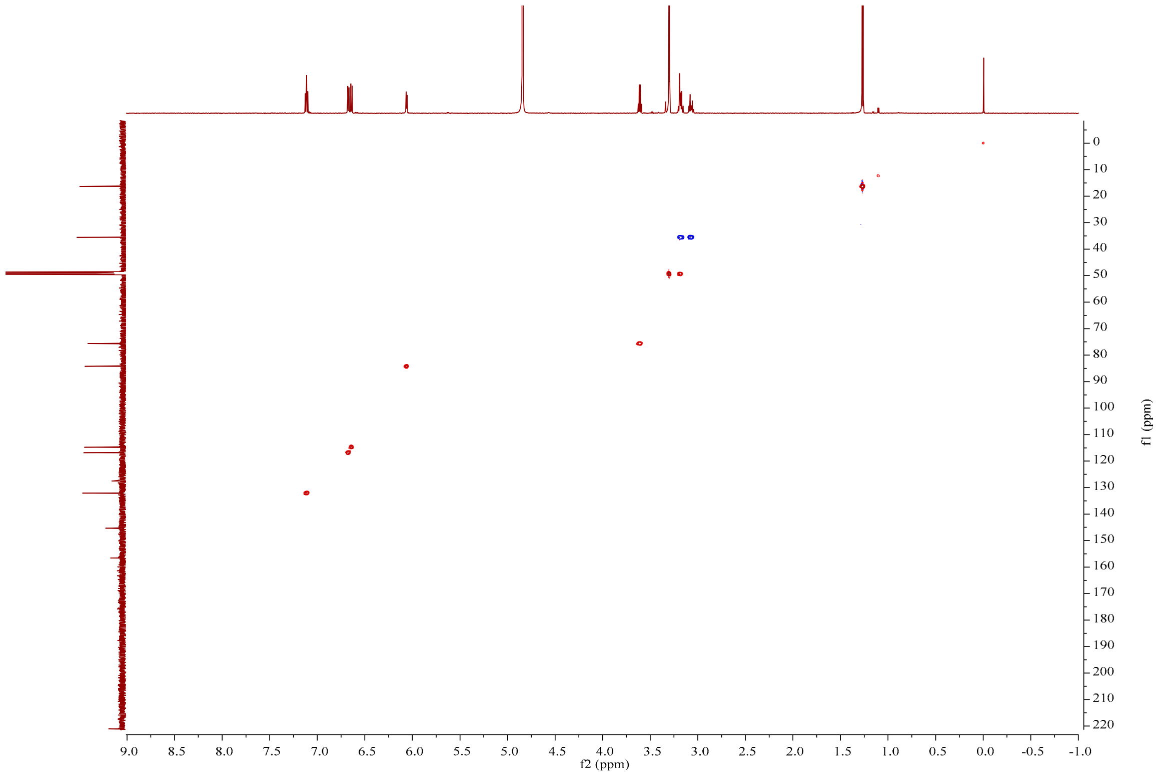

Figure S3e HSQC spectrum (150 MHz/600 MHz, CD_3OD) of **2**.

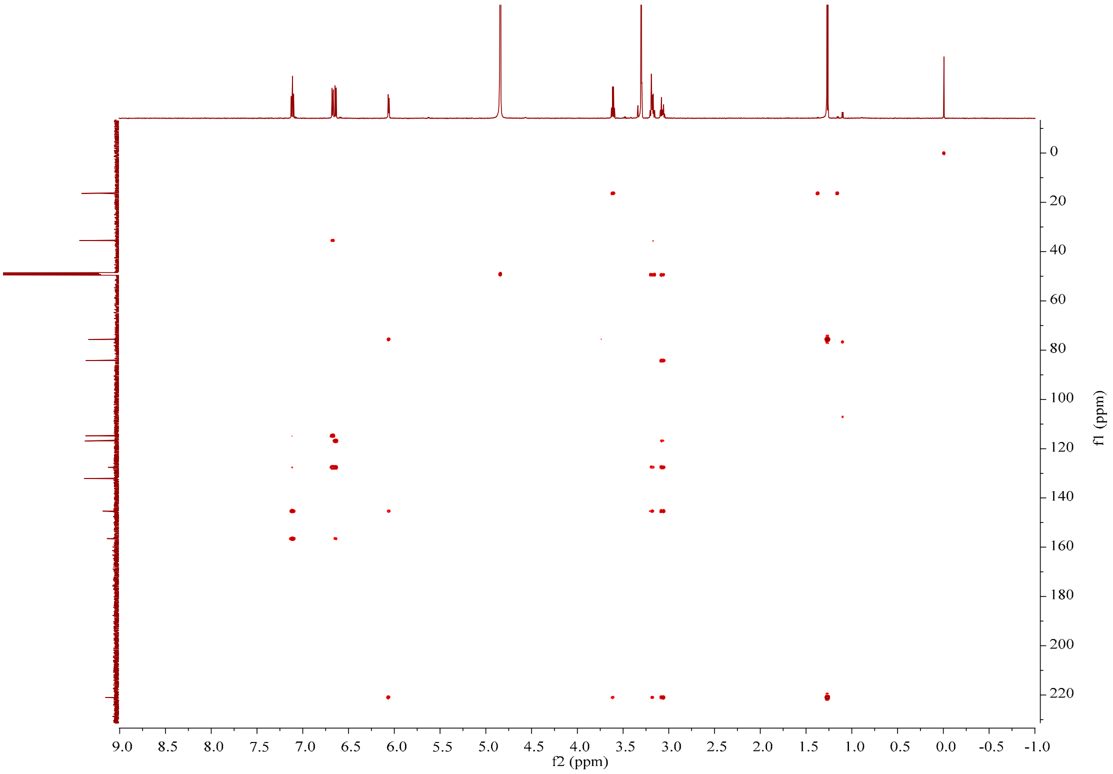

Figure S3f HMBC spectrum (150 MHz/600 MHz, CD_3OD) of **2**.

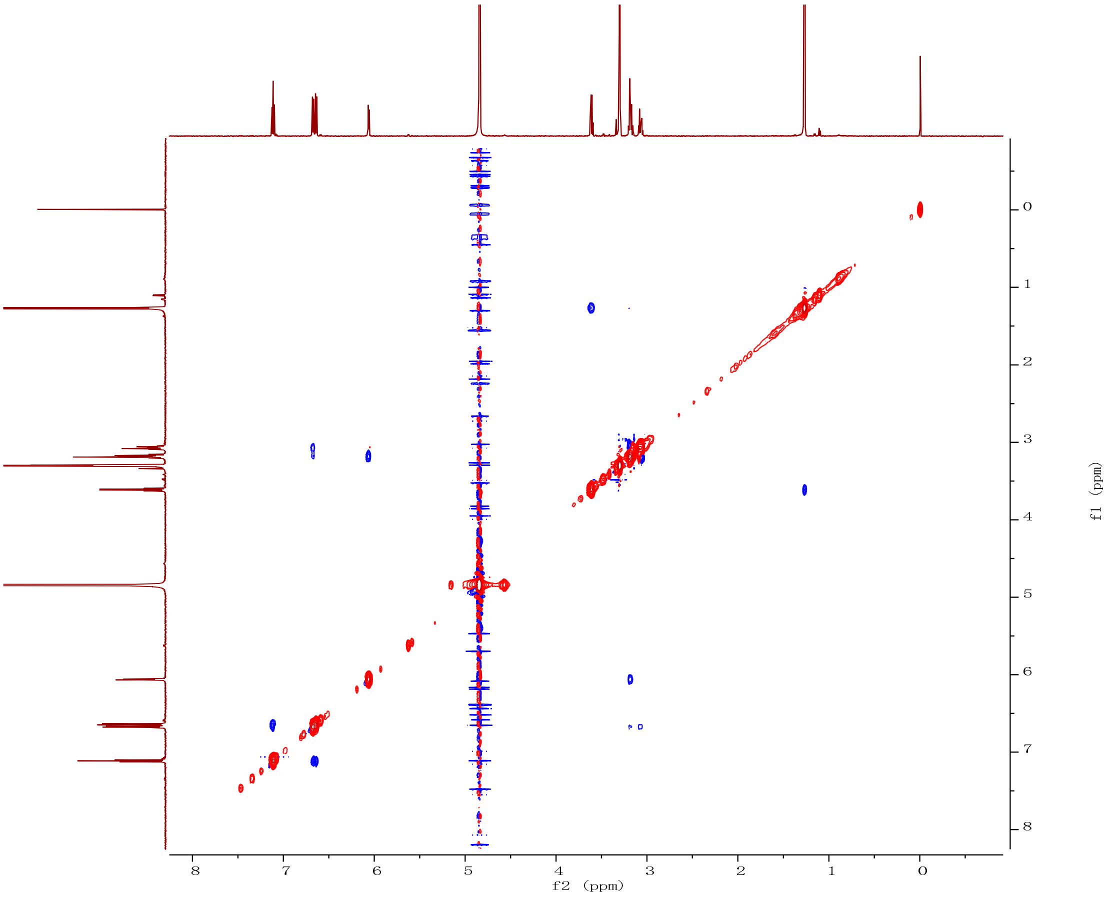

Figure S3g NOESY spectrum (600 MHz, CD_3OD) of **2**.

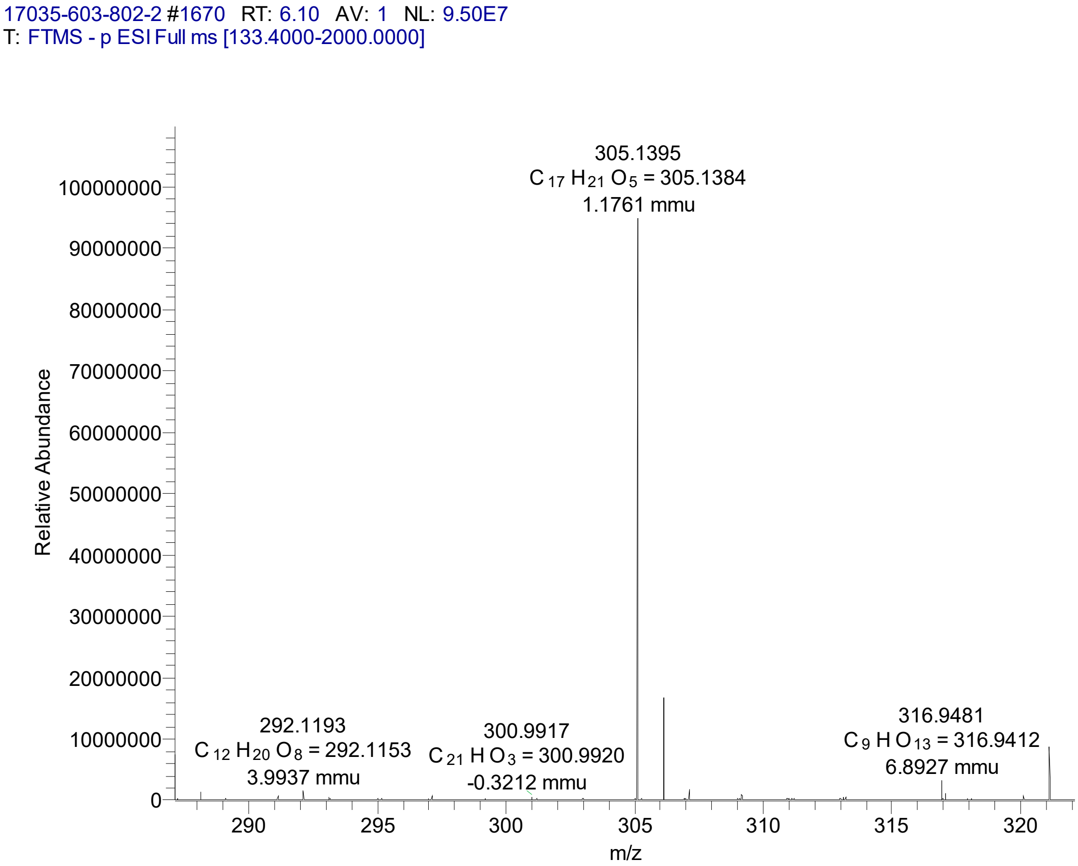

Figure S4a HRESIMS spectrum of **3**.

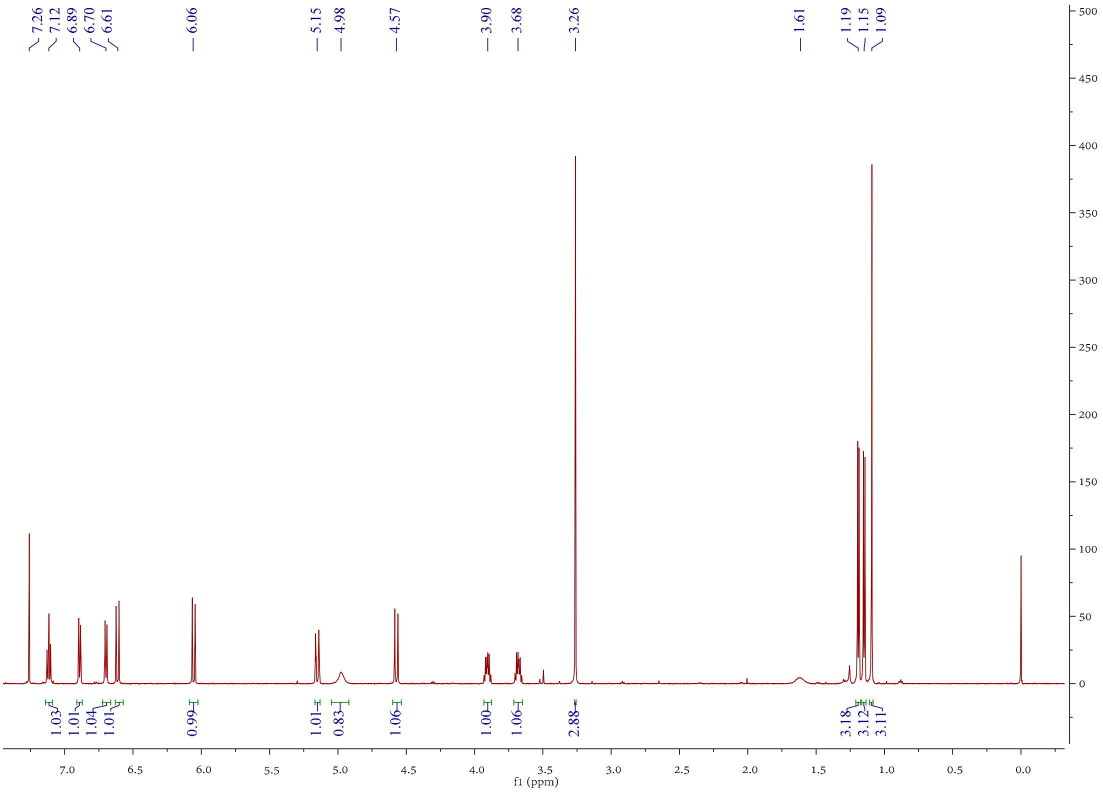

Figure S4b ^{1}H NMR spectrum (600 MHz, $CDCl_3$) of **3**.

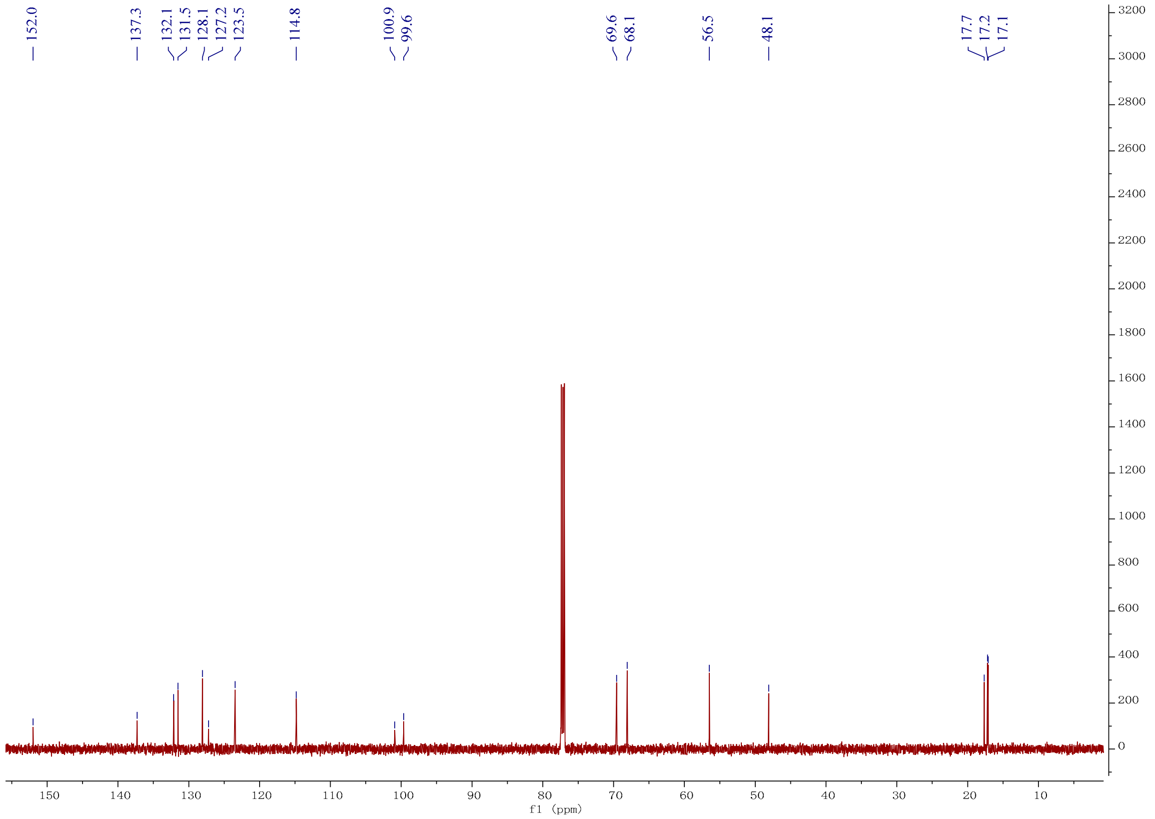

Figure S4c ^{13}C NMR spectrum (150 MHz, $CDCl_3$) of **3**.

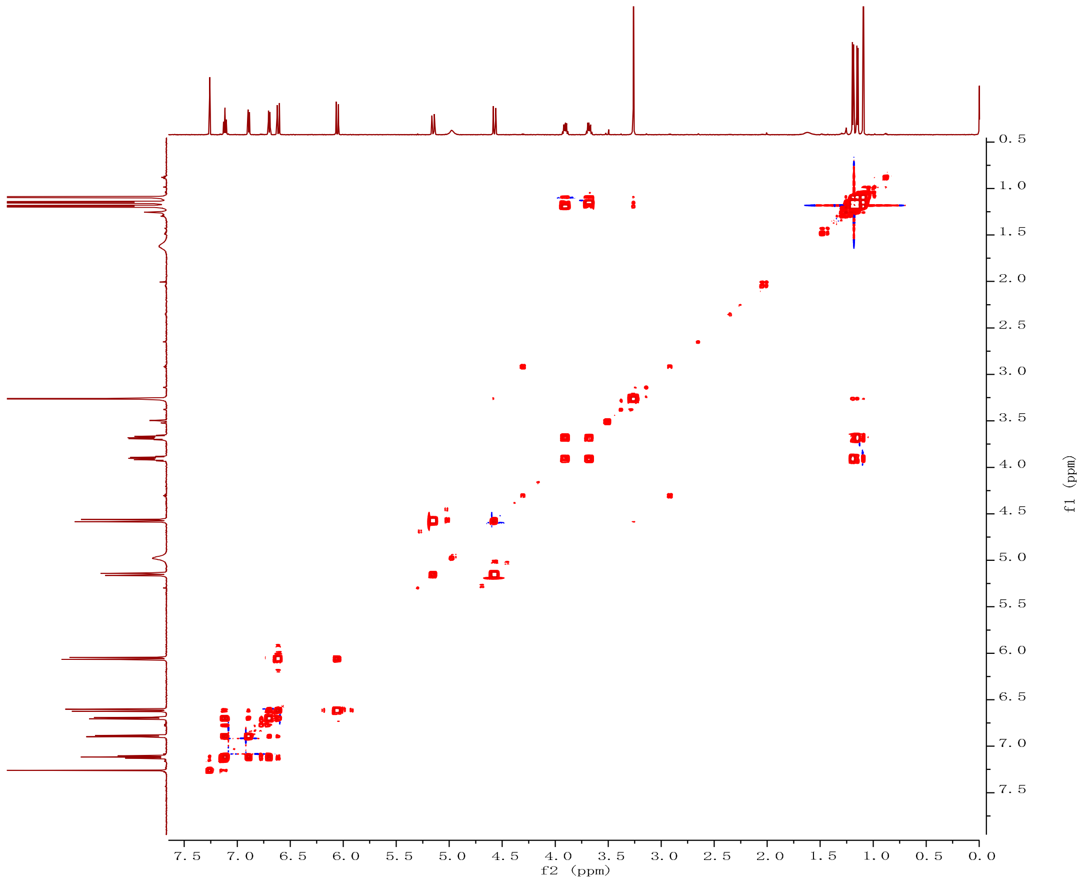

Figure S4d ^{1}H-^{1}H COSY spectrum (600 MHz, $CDCl_3$) of **3**.

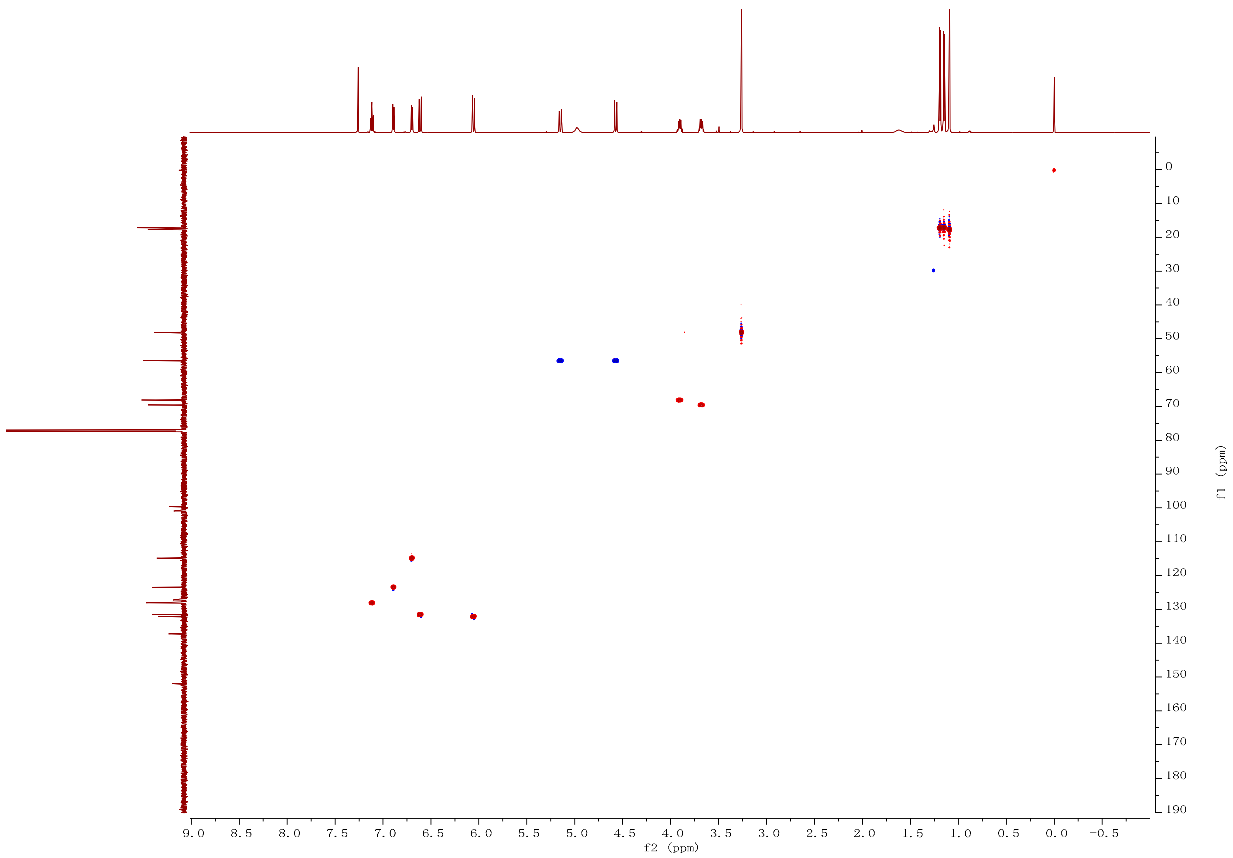

Figure S4e HSQC spectrum (150 MHz/600 MHz, $CDCl_3$) of **3**.

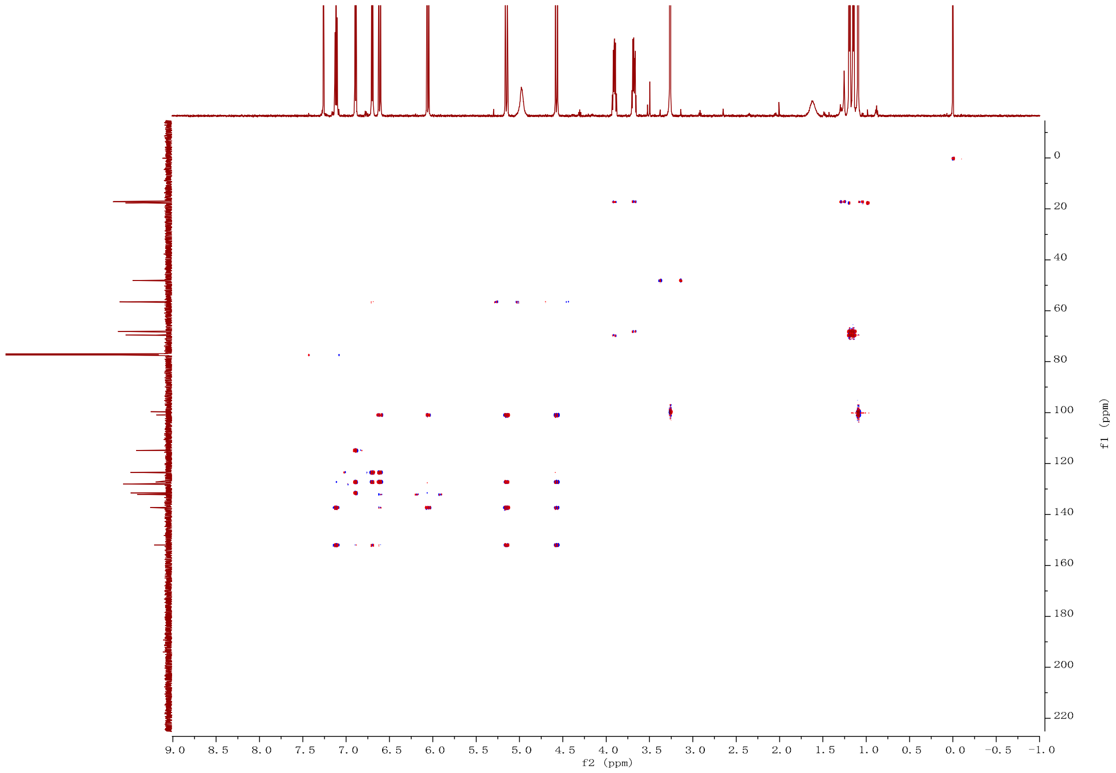

Figure S4f HMBC spectrum (150 MHz/600 MHz, $CDCl_3$) of **3**.

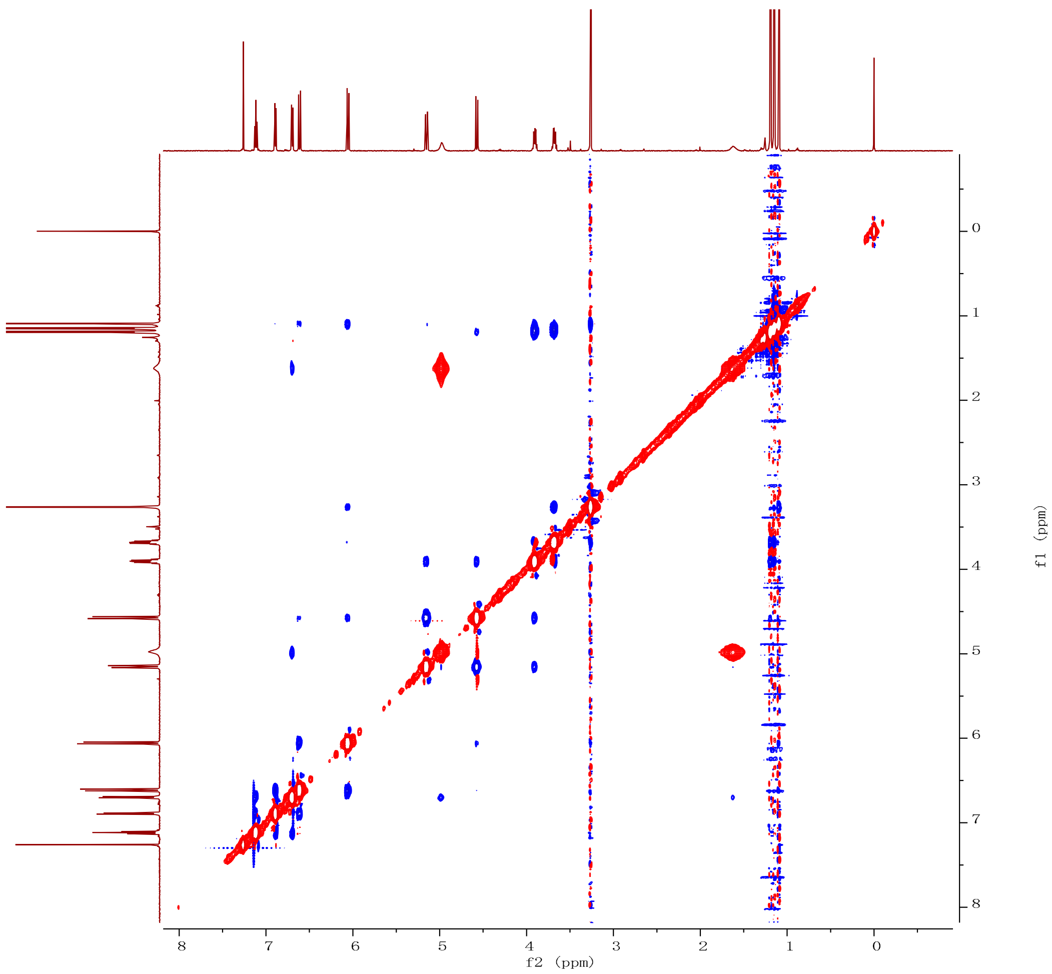

Figure S4g NOESY spectrum (600 MHz, $CDCl_3$) of **3**.

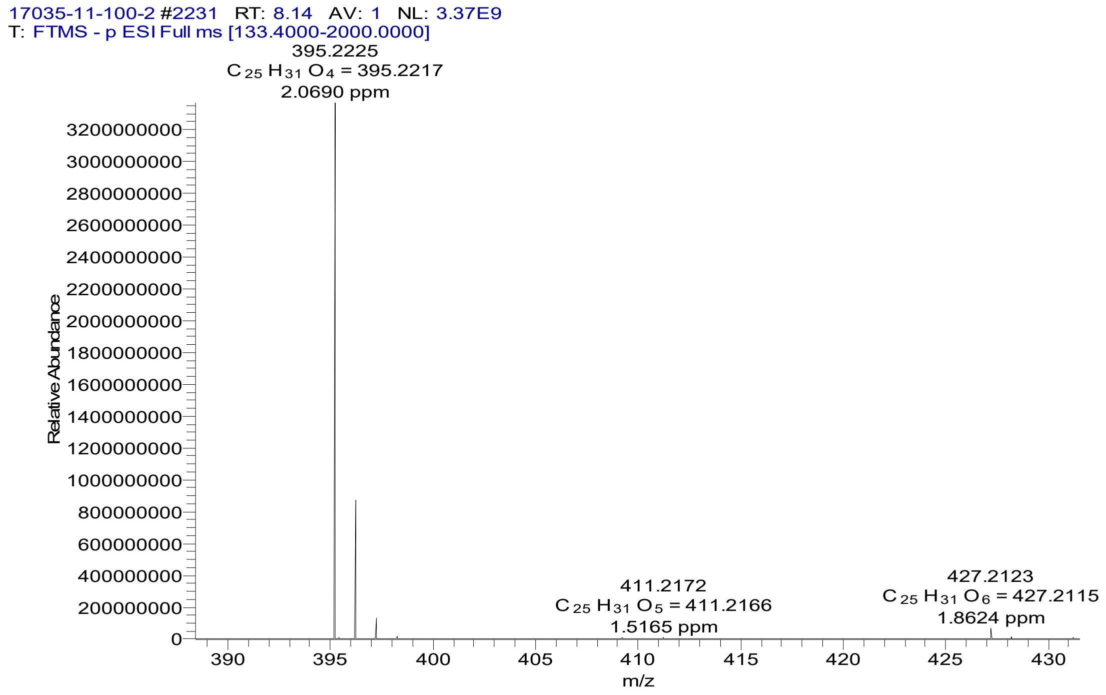

Figure S5a HRESIMS spectrum of **4**.

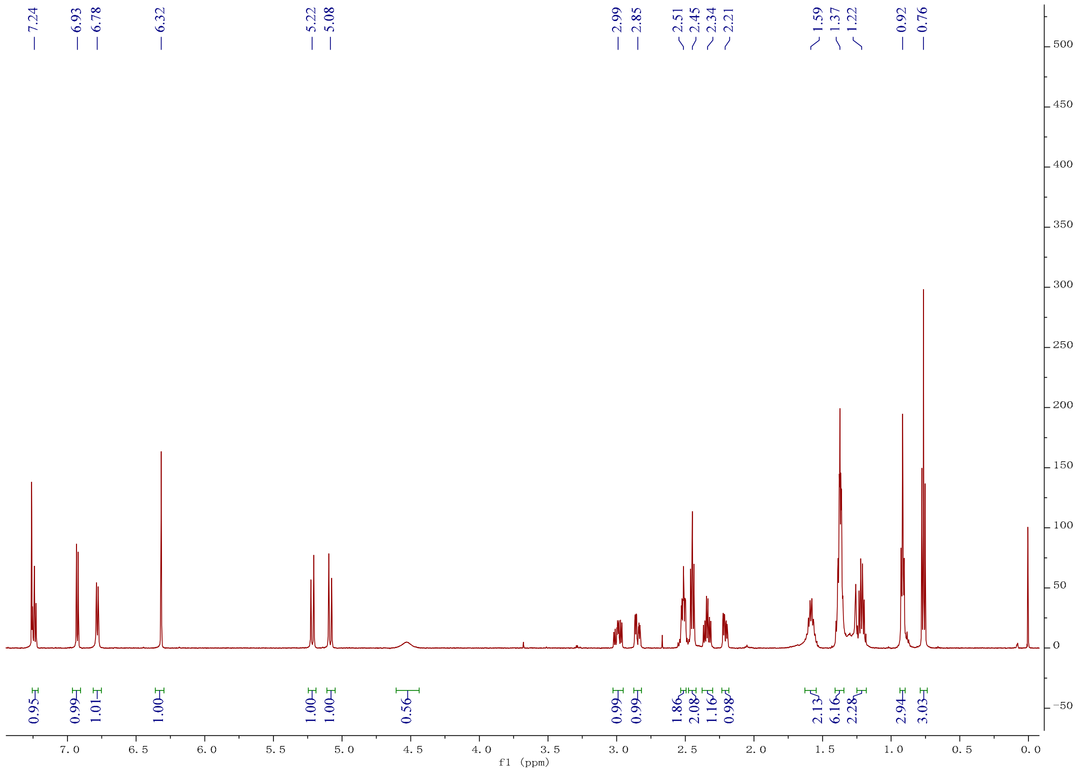

Figure S5b ^{1}H NMR spectrum (600 MHz, CDCl3) of **4**.

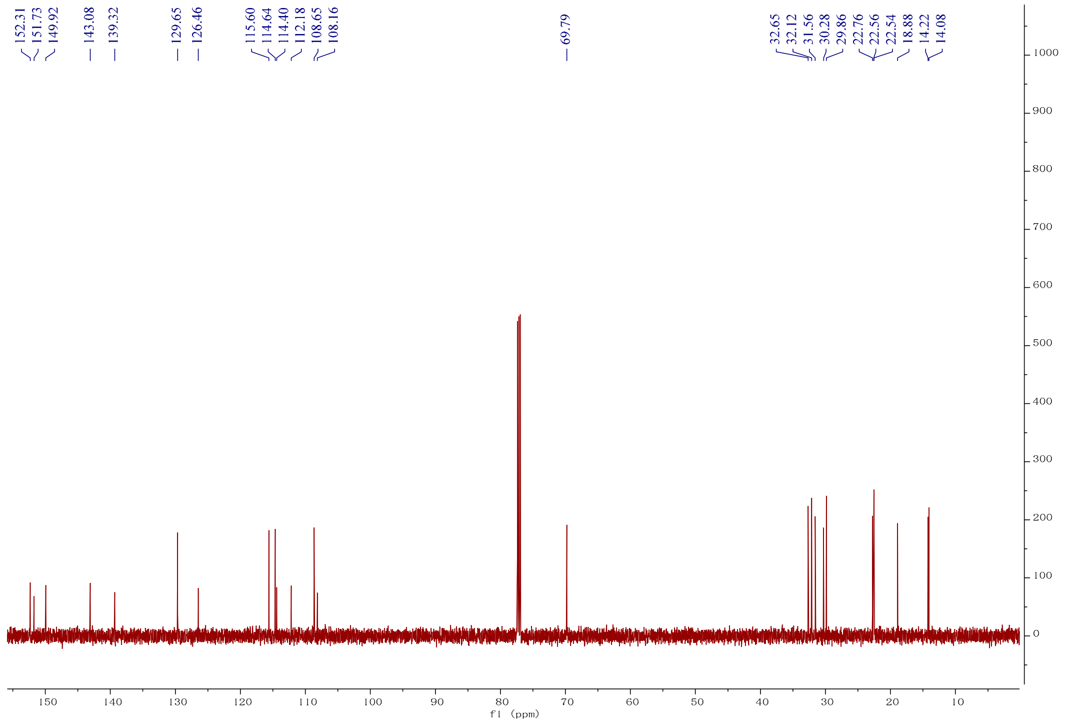

Figure S5c ^{13}C NMR spectrum (150 MHz, CDCl3) of **4**.

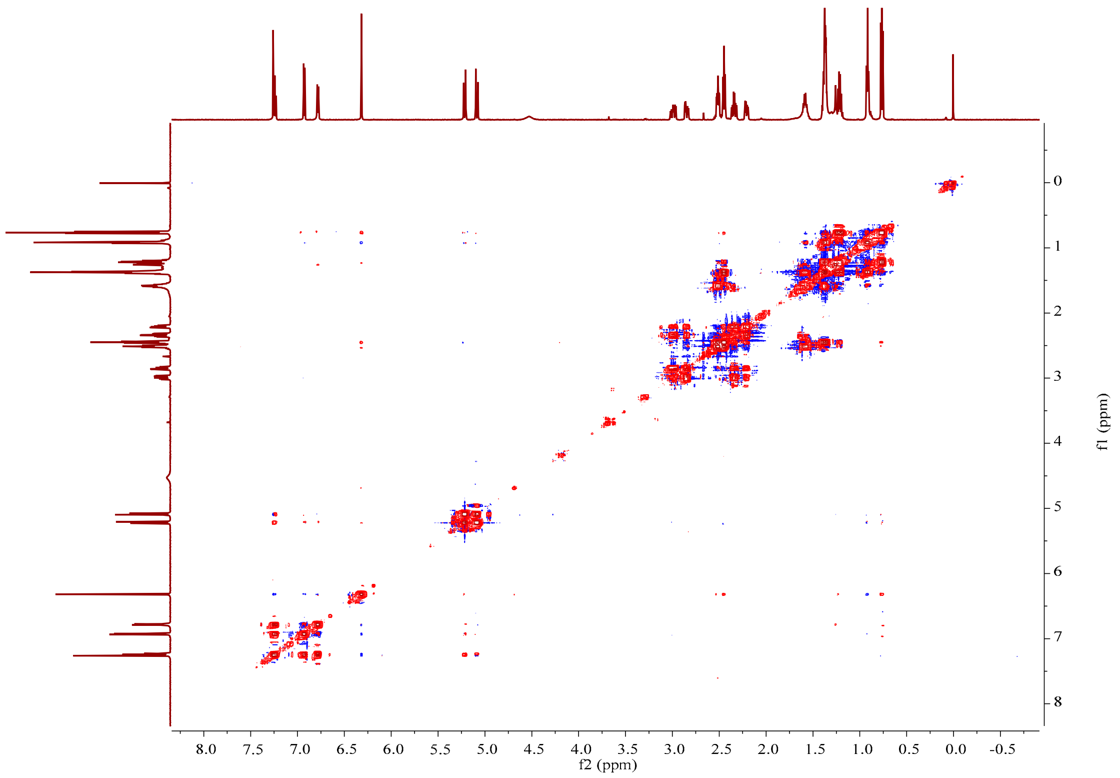

Figure S5d ^{1}H-^{1}H COSY spectrum (600 MHz, $CDCl_3$) of **4**.

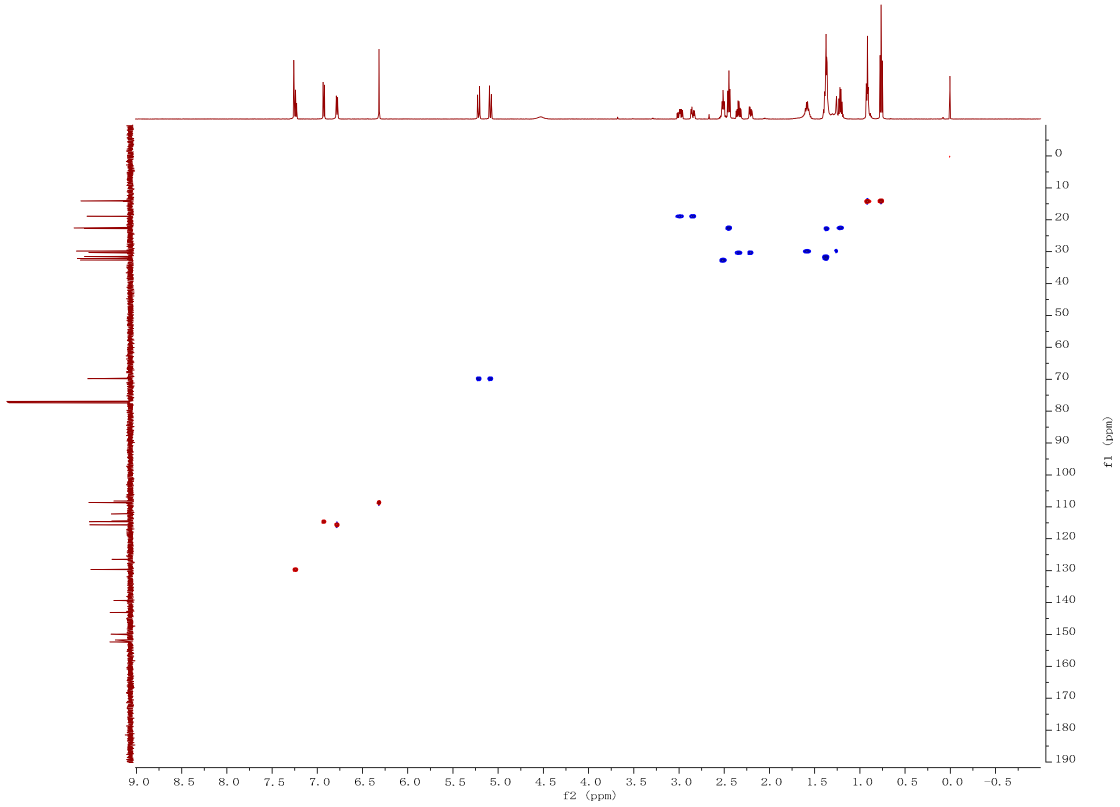

Figure S5e HSQC spectrum (150 MHz/600 MHz, $CDCl_3$) of **4**.

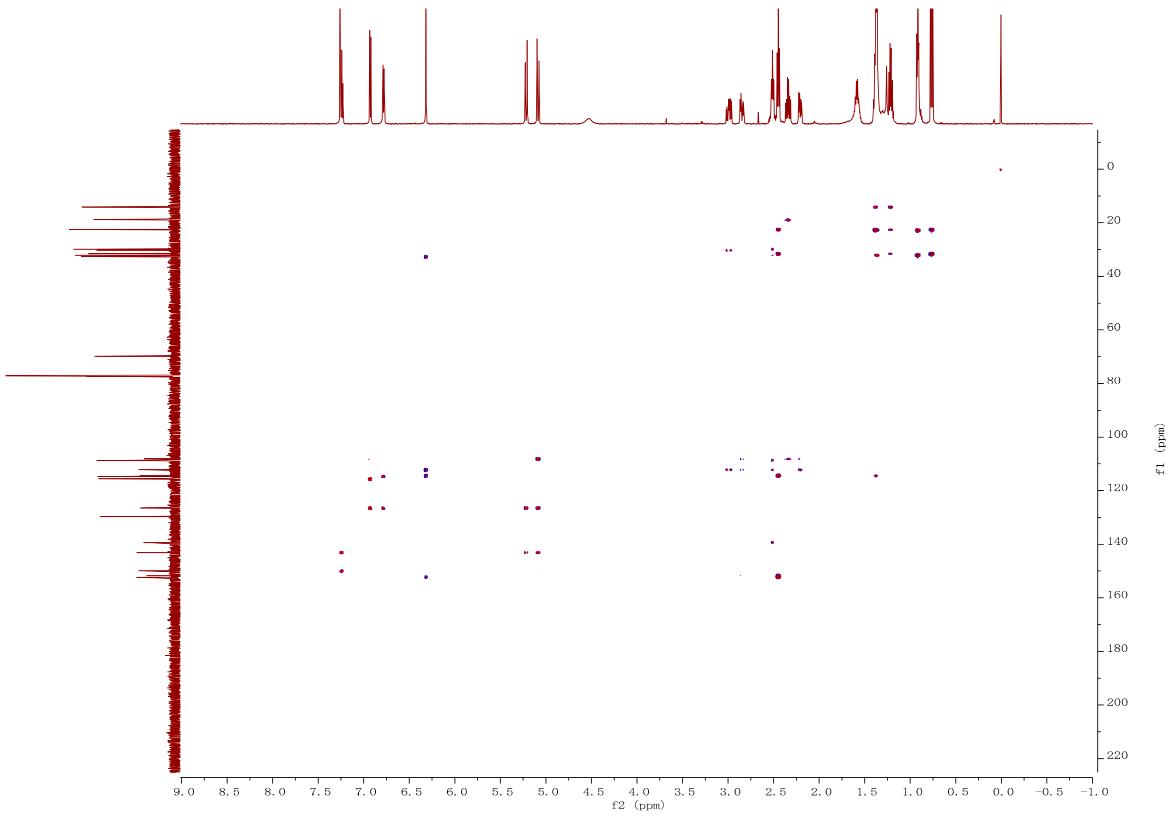

Figure S5f HMBC spectrum (150 MHz/600 MHz, $CDCl_3$) of **4**.

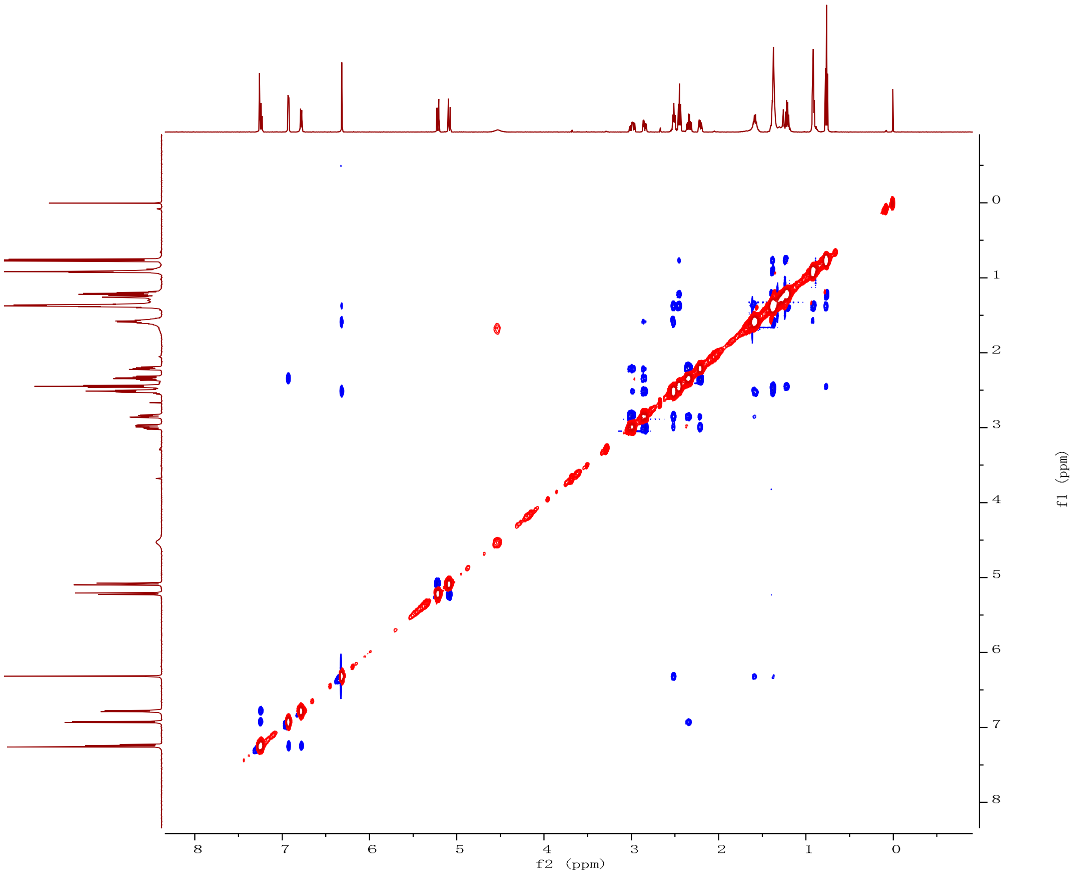

Figure S5g NOESY spectrum (600 MHz, $CDCl_3$) of **4**.

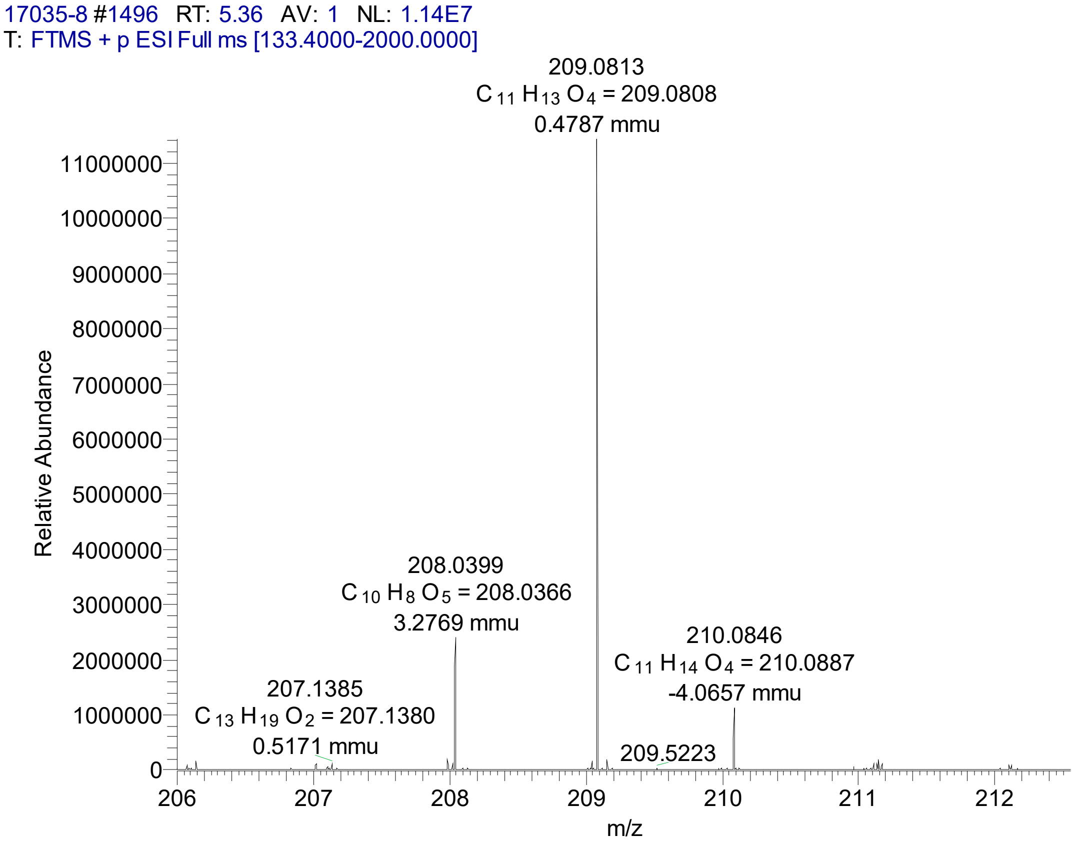

Figure S6a HRESIMS spectrum of **5**.

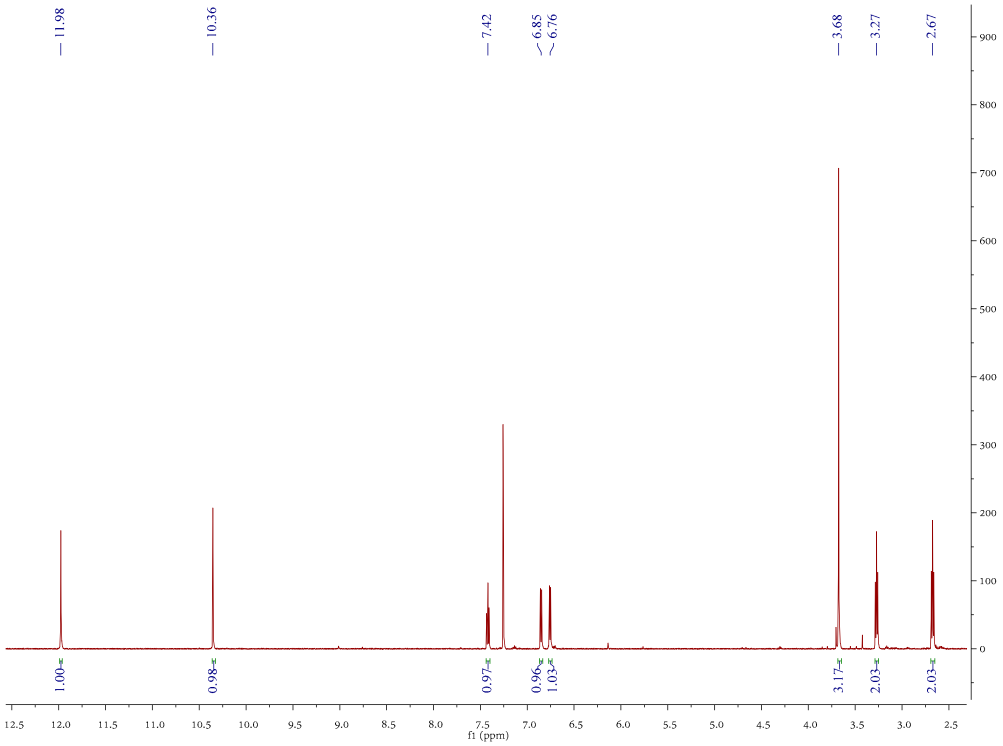

Figure S6b ^{1}H NMR spectrum (600 MHz, $CDCl_3$) of **5**.

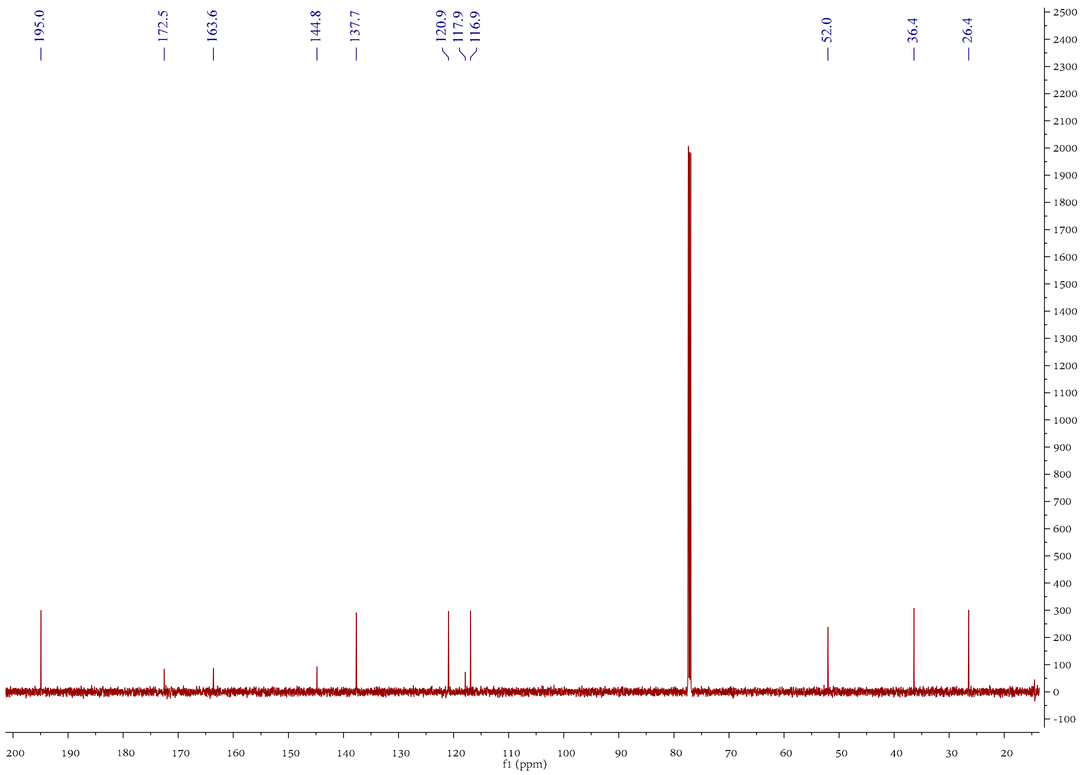

Figure S6c ^{13}C NMR spectrum (150 MHz, $CDCl_3$) of **5**.

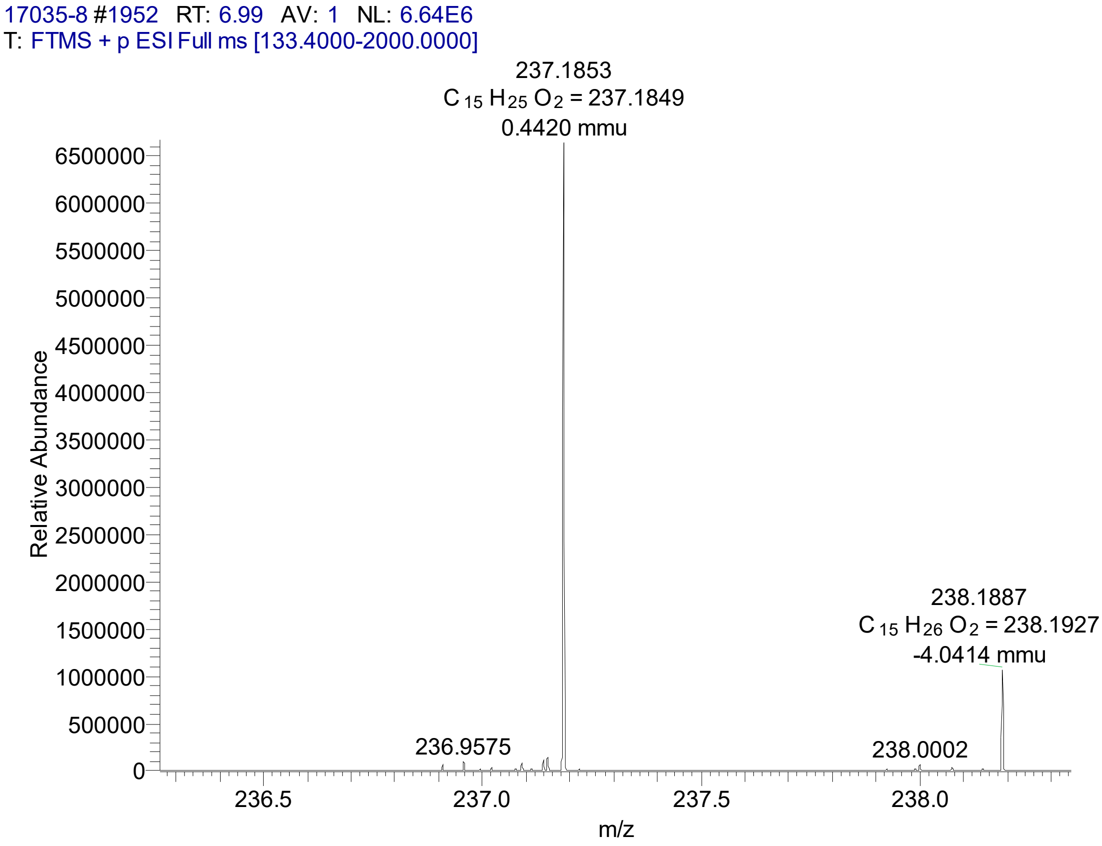

Figure S7a HRESIMS spectrum of **6**.

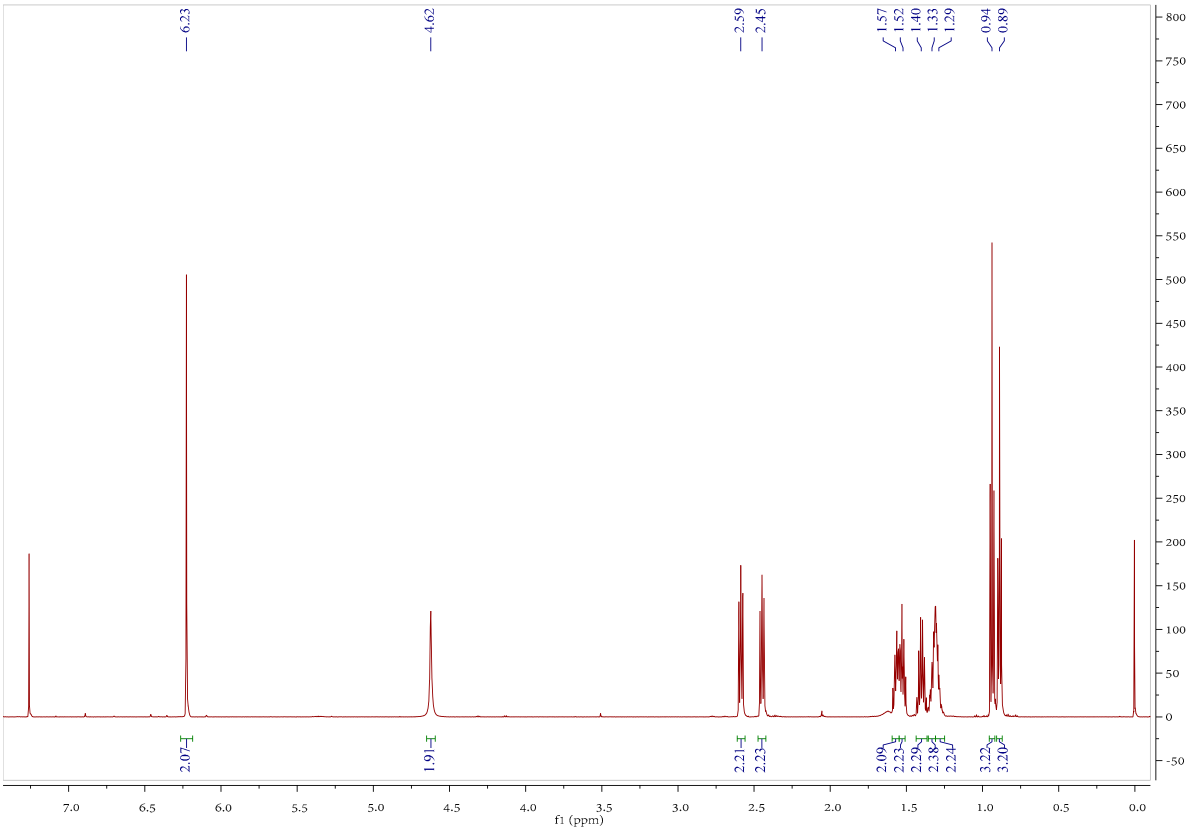

Figure S7b ^{1}H NMR spectrum (600 MHz, $CDCl_3$) of **6**.

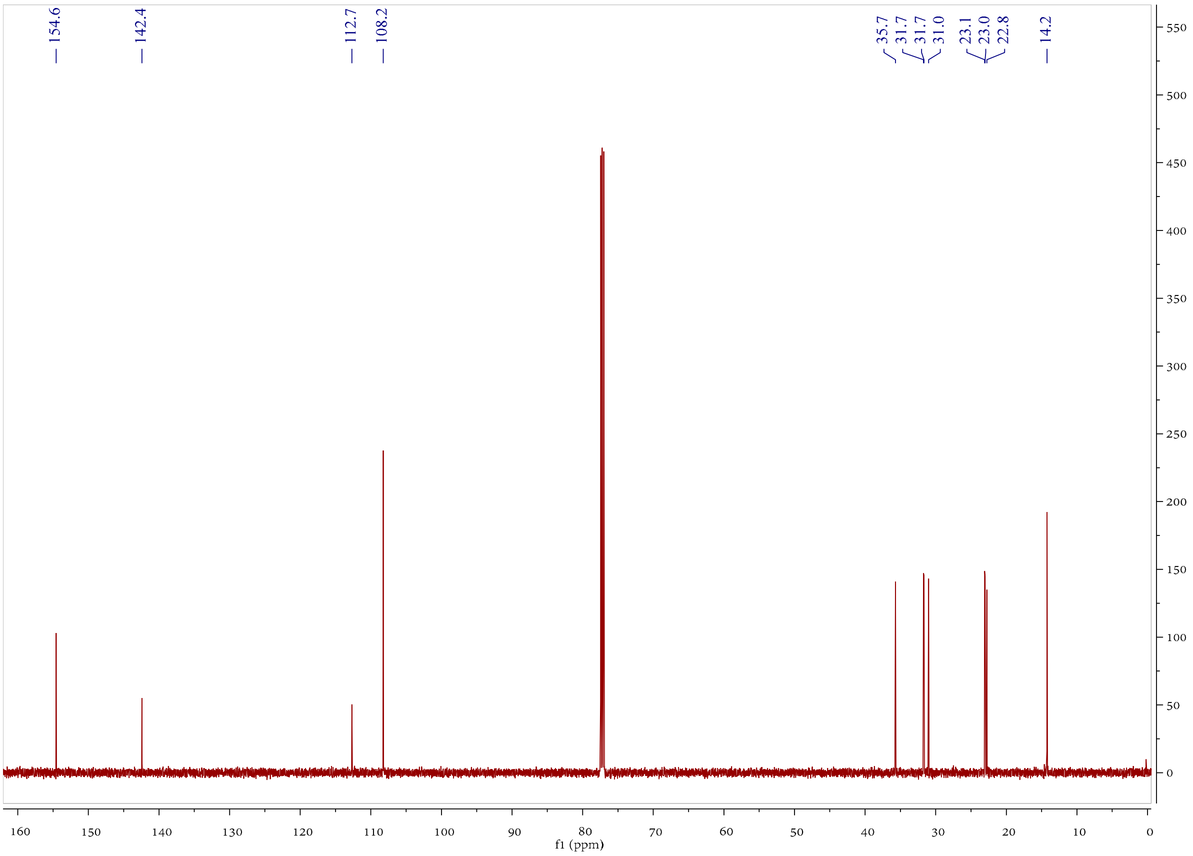

Figure S7c ^{13}C NMR spectrum (150 MHz, $CDCl_3$) of **6**.

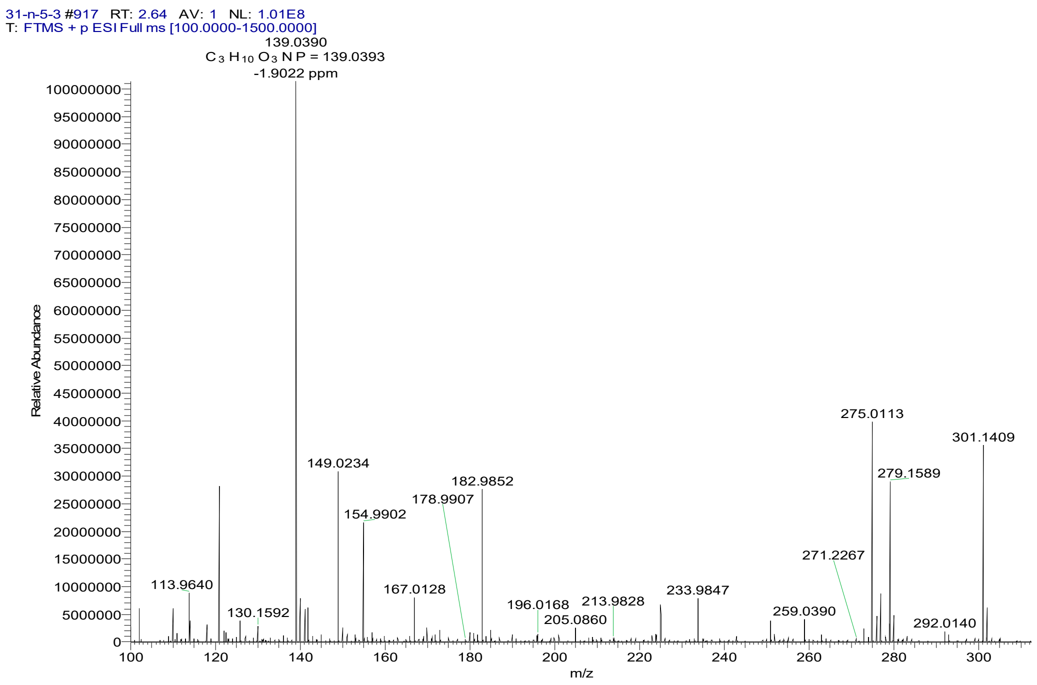

Figure S8a HRESIMS spectrum of **7**.

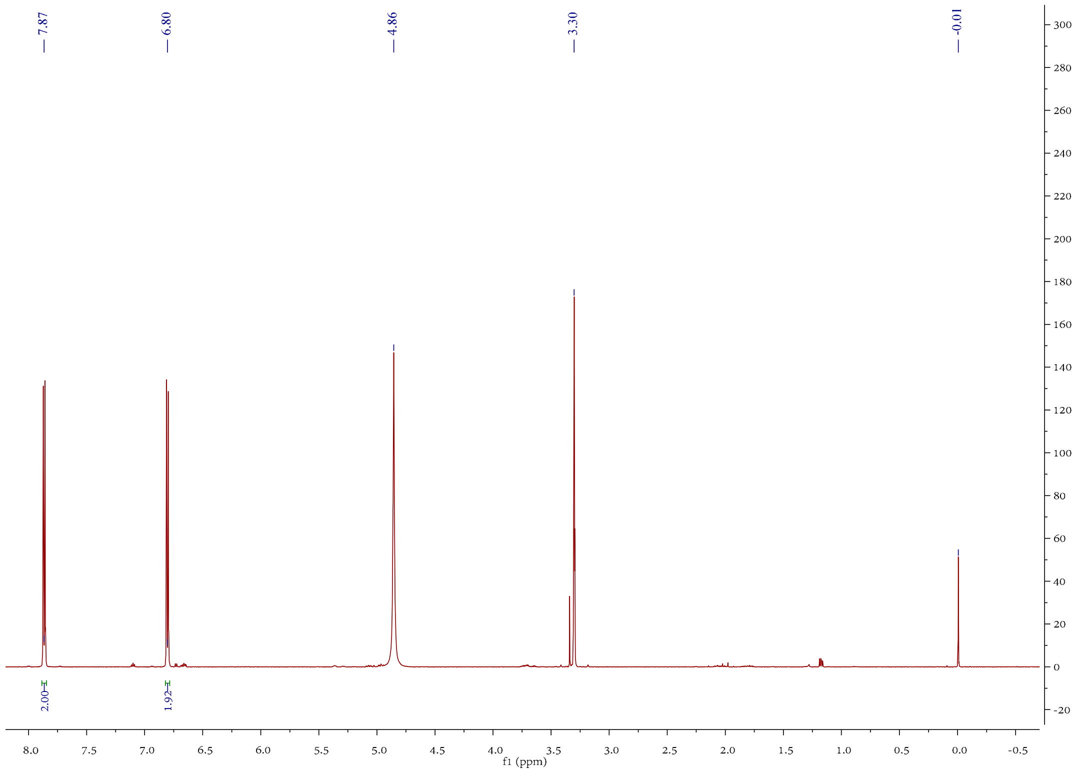

Figure S8b ^{1}H NMR spectrum (600 MHz, CD_3OD) of **7**.

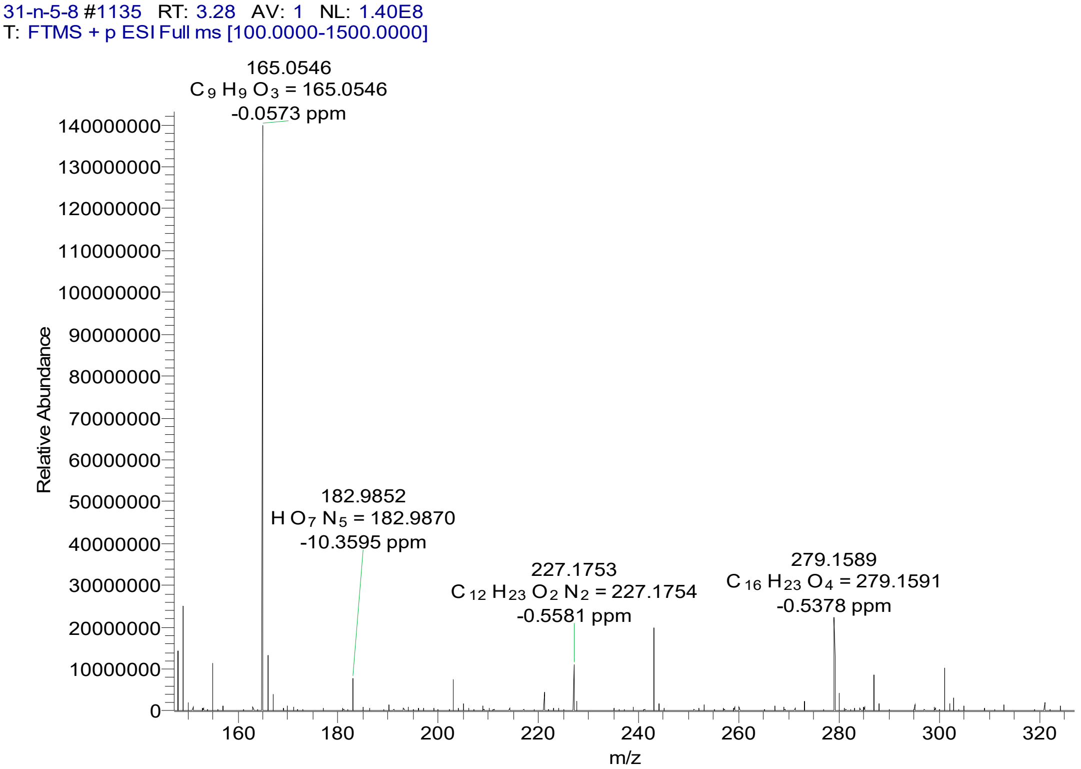

Figure S9a HRESIMS spectrum of **8**.

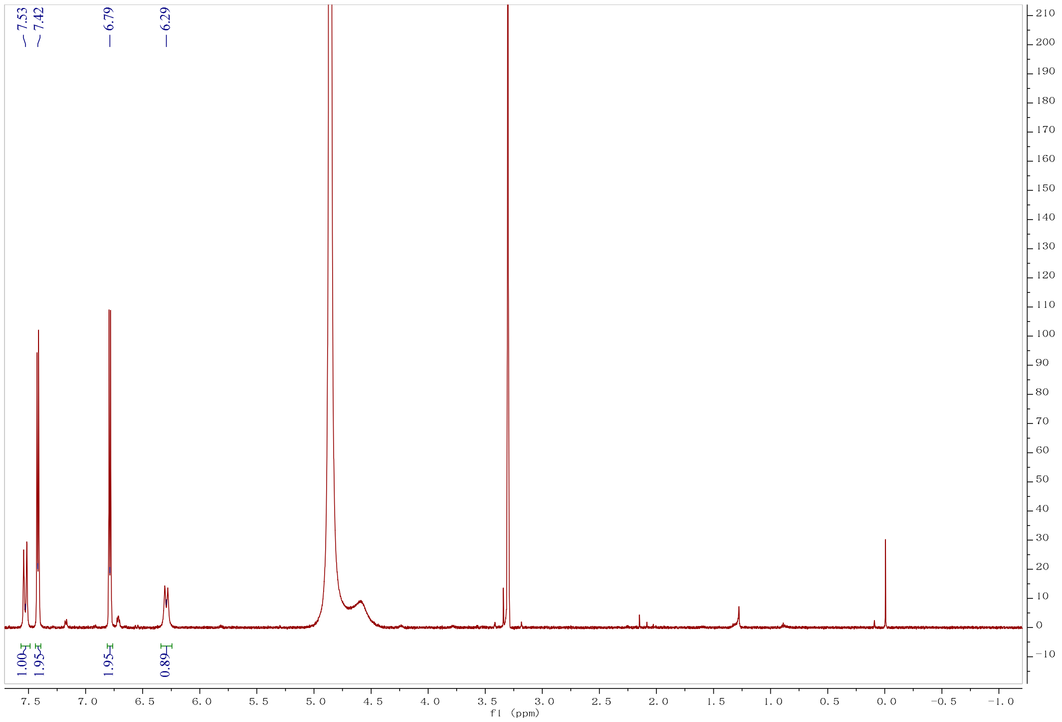

Figure S9b ^{1}H NMR spectrum (600 MHz, CD_3OD) of **8**.

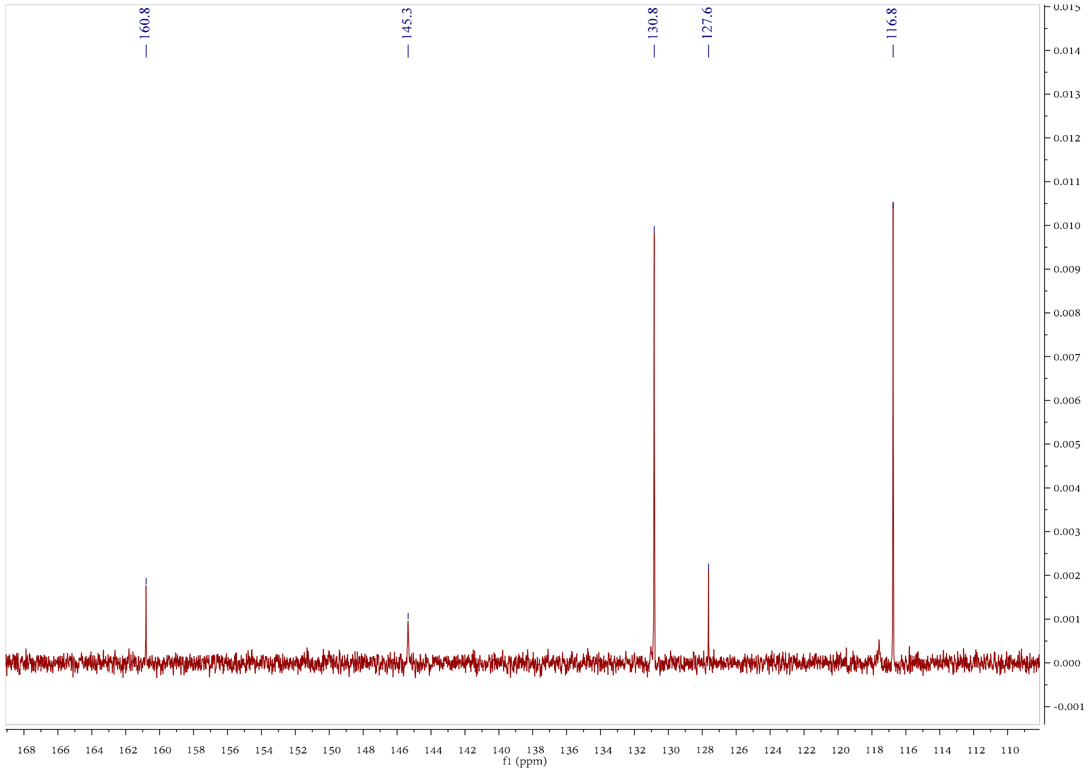

Figure S9c ^{13}C NMR spectrum (150 MHz, CD_3OD) of **8**.

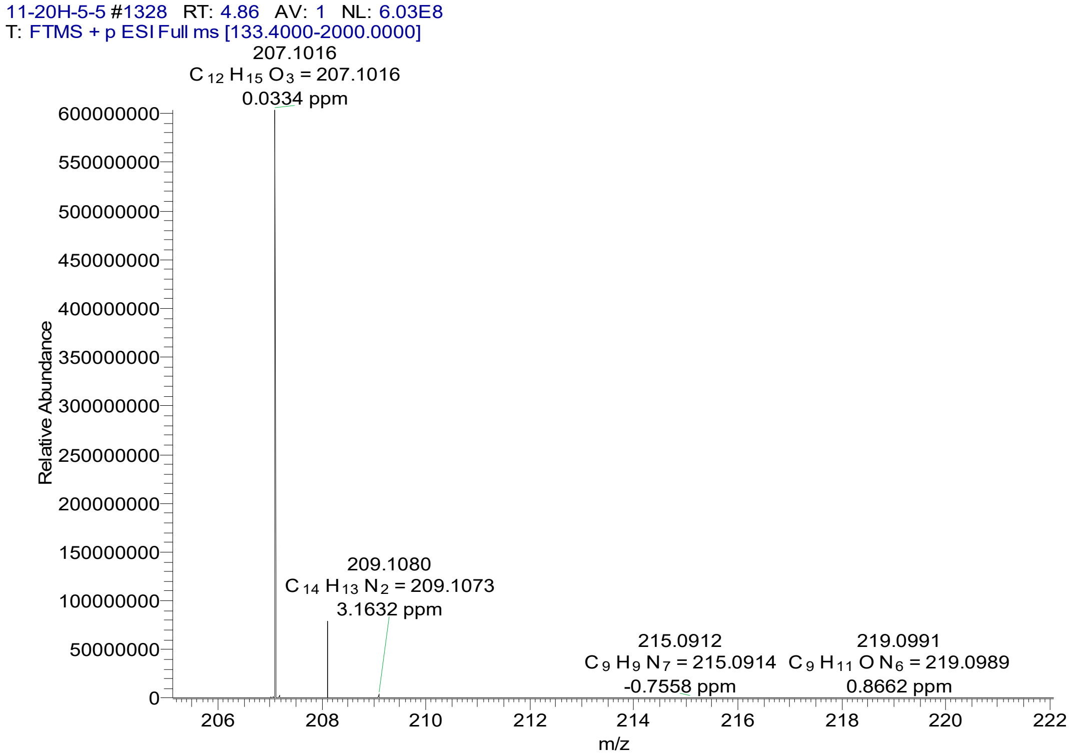

Figure S10a HRESIMS spectrum of **9**.

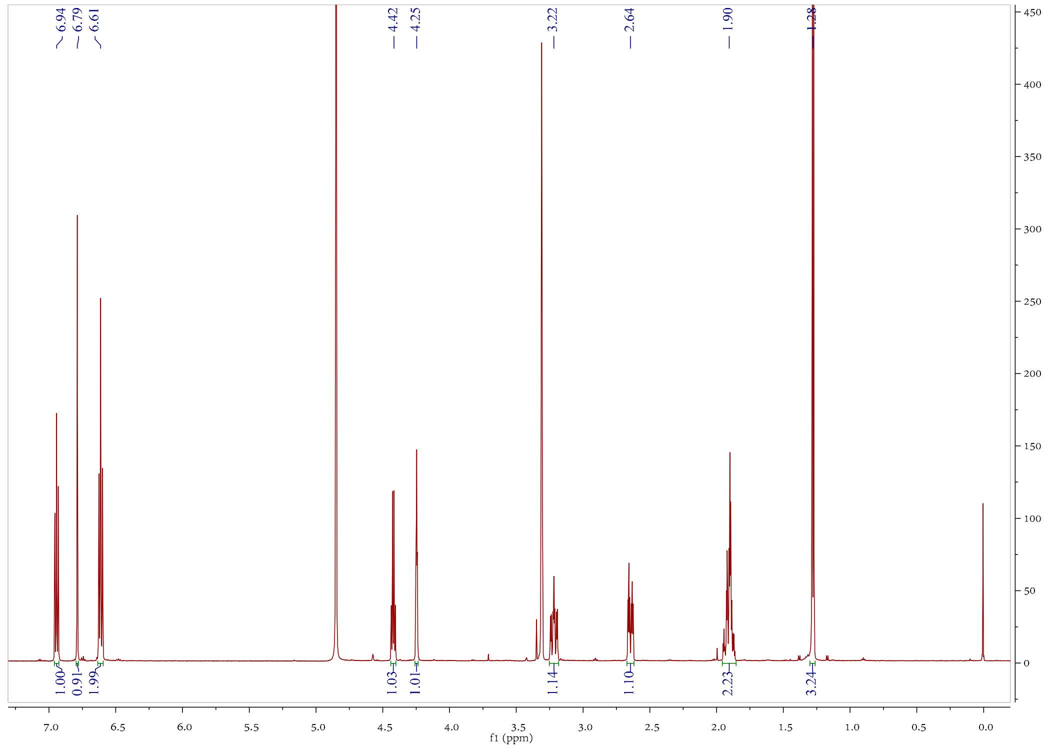

Figure S10b ^{1}H NMR spectrum (600 MHz, CD_3OD) of **9**.

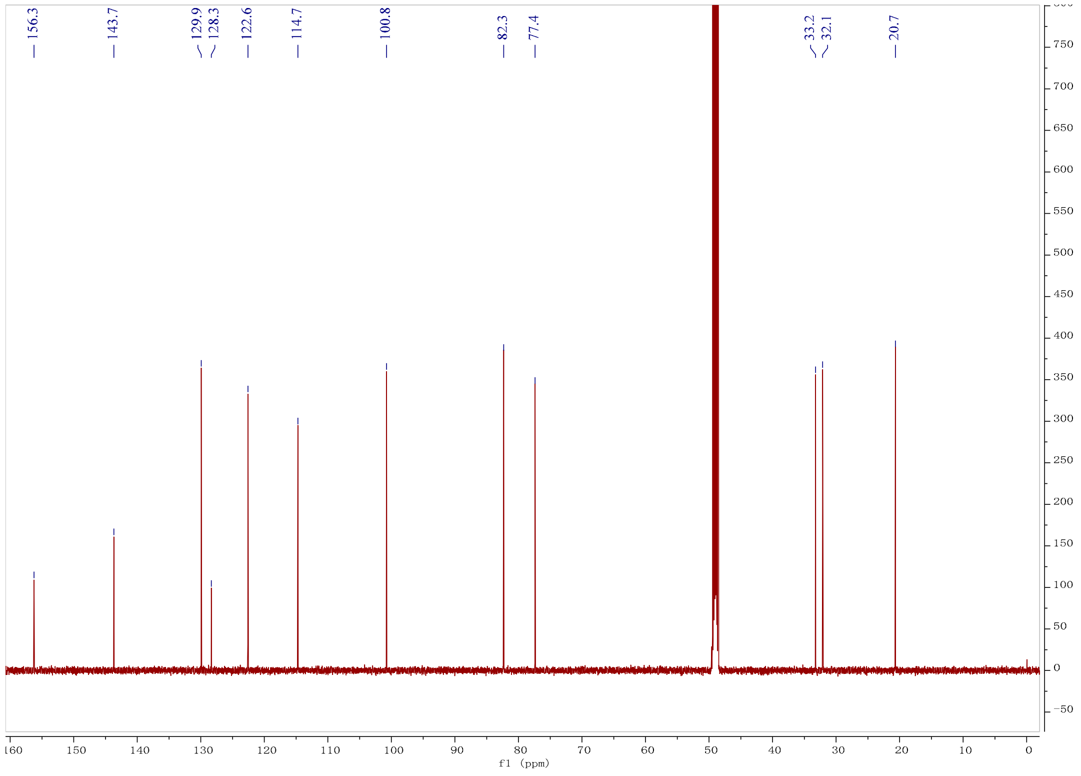

Figure S10c ^{13}C NMR spectrum (150 MHz, CD_3OD) of **9**.

Supplementary Tables

Table S1. Composition of the culture media.

media	component
T7 medium	Malt Extract 10 g/L, Yeast Extract 10 g/L, $(NH_4)_2SO_4$ 10 g/L, KH_2PO_4 1 g/L, Rice 20 g/L, Starch 10 g/L
T1 medium	Malt Extract 10 g/L, Yeast Extract 10 g/L, $(NH_4)_2SO_4$ 10 g/L, KH_2PO_4 1 g/L, Rice 80 g/L
Malt Extract medium (ME)	Malt Extract 10 g/L, Peptone 5 g/L
Potato Dextrose Broth (PDB)	Potato starch 4 g/L, Dextrose 20 g/L
Potato Dextrose Agar (PDA)	Potato starch 4 g/L, Dextrose 20 g/L, Agar 15 g/L
ISP2	Malt Extract 10 g/L, Yeast Extract 4 g/L, Glucose 4 g/L
Czapek's medium (CHASHI)	$NaNO_3$ 2 g/L, K_2HPO_4 1 g/L, $MgSO_4.7H_2O$ 0.5 g/L, KCl 0.5 g/L, $FeSO_4$ 0.01 g/L, Sucrose 30 g/L
starch	soluble starch 10 g/L, Peptone 1 g/L
GPY	Yeast Extract 10 g/L, Peptone 20 g/L, Glucose 20 g/L
Z4 medium	Mannitol 20 g/L, Glucose 20 g/L, Peptone 10 g/L, Yeast Extract 10 g/L, K_2HPO_4 0.5 g/L, $MgSO_4.7H_2O$ 0.3 g/L
Z5 medium	Malt Extract 3 g/L, Yeast Extract 3 g/L, Glucose 20 g/L Peptone 10 g/L
Rice medium	Rice 80 g/120 mL

Table S2. DP4 probability of C NMR chemical shifts of **4a** (8*R*) and **4b** (8*S*).

No.	δexp	δcal		δscal		corrected error($\Delta\delta$)		t distribution		Probability	
		4a	4b	4a	4b	4a	4b	4a	4b	4a	4b
1	69.8	74.2	74.1	69.2	63.5	0.6	6.3	0.60	0.99	0.40	0.01
2	126.5	133.5	133.3	127.4	117.3	-0.9	9.2	0.65	1.00	0.35	0.00
3	149.9	157.8	157.9	151.3	139.6	-1.4	10.3	0.72	1.00	0.28	0.00
4	115.6	116.9	116.9	111.2	102.4	4.4	13.2	0.96	1.00	0.04	0.00
5	129.7	134.1	134.2	128.0	118.1	1.7	11.6	0.76	1.00	0.24	0.00
6	114.6	119.0	119.1	113.2	104.4	1.4	10.2	0.73	1.00	0.27	0.00
7	143.1	151.7	151.7	145.2	133.9	-2.1	9.2	0.81	1.00	0.19	0.00
8	108.2	114.9	114.9	109.2	100.5	-1.0	7.7	0.67	1.00	0.33	0.00
9	30.3	34.2	34.1	29.9	27.3	0.4	3.0	0.57	0.89	0.43	0.11
1′	18.9	22.7	22.6	18.6	16.8	0.3	2.1	0.54	0.81	0.46	0.19
2′	112.2	116.2	117.0	110.5	102.4	1.7	9.8	0.76	1.00	0.24	0.00
3′	151.7	159.8	160.6	153.2	142.0	-1.5	9.7	0.74	1.00	0.26	0.00
4′	114.4	117.9	121.4	112.2	106.5	2.2	7.9	0.82	1.00	0.18	0.00
5′	152.3	161.1	160.6	154.5	142.1	-2.2	10.2	0.82	1.00	0.18	0.00
6′	108.6	111.9	110.0	106.3	96.2	2.3	12.4	0.83	1.00	0.17	0.00
7′	139.3	146.7	145.8	140.4	128.6	-1.1	10.7	0.68	1.00	0.32	0.00
8′	32.7	38.2	39.0	33.9	31.7	-1.2	1.0	0.69	0.67	0.31	0.33
9′	29.9	36.7	37.5	32.4	30.3	-2.5	-0.4	0.85	0.57	0.15	0.43
10′	32.1	37.4	38.3	33.1	31.0	-1.0	1.1	0.67	0.68	0.33	0.32
11′	22.8	28.4	28.7	24.2	22.3	-1.4	0.5	0.73	0.58	0.27	0.42
12′	14.2	15.4	16.0	11.5	10.8	2.7	3.4	0.87	0.92	0.13	0.08
13′	22.6	27.7	27.8	23.5	21.5	-0.9	1.1	0.65	0.67	0.35	0.33
14′	31.6	36.5	36.5	32.2	29.4	-0.6	2.2	0.60	0.82	0.40	0.18
15′	22.5	28.4	28.7	24.3	22.3	-1.8	0.2	0.77	0.53	0.23	0.47
16′	14	16.0	16.2	12.1	11.0	1.9	3.0	0.79	0.89	0.21	0.11
Product of probabilities										5.37E-16	3.53E-52
Bayes's theorem probability (%)										100	0

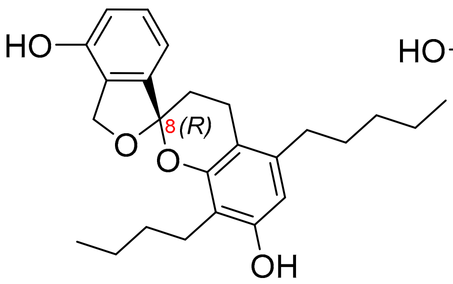

4a

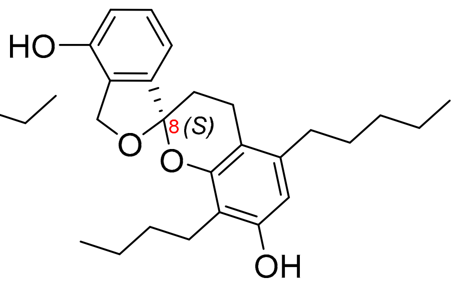

4b

Table S3. Cytotoxic activity of **1−9**

Cell	Cell inhibition (%) (10 μM)									
	**1**	**2**	**3**	**4**	**5**	**6**	**7**	**8**	**9**	Doxorubicin
A549	4	2	9	13	0	17	0	5	5	71
MKN-45	-8	18	21	36	8	10	12	6	20	78
HCT 116	18	-7	-8	1	8	15	9	10	8	84
HeLa	11	6	6	-13	3	7	4	3	6	91
K-562	-1	5	7	21	8	4	4	15	2	85
786-O	11	16	16	18	3	8	3	0	3	88
TE-1	15	-2	1	8	-2	39	7	5	11	81
5637	12	4	7	10	-7	-3	0	1	4	94
GBC-SD	2	-8	3	14	3	10	6	4	5	82
MCF7	-4	6	1	17	-5	19	-4	1	7	47
HepG2	11	0	1	4	-4	0	3	6	4	72
SF126	5	6	5	9	4	18	-3	1	-3	69
DU145	8	6	5	9	2	21	4	9	6	67
CAL-62	16	-3	-3	0	-12	-2	7	15	10	73
PATU8988T	7	-2	-3	-2	-2	-1	9	10	6	87
HOS	6	-6	-3	5	11	3	3	5	4	86
A-375	22	-1	1	5	22	18	16	28	24	77
A-673	-1	4	7	5	3	6	-6	-6	-3	72
L-02	7	-4	-3	1	3	7	5	10	0	81
293T	28	-3	9	10	-3	19	8	36	16	70
